# Parkin coregulates glutathione metabolism in adult mammalian brain

**DOI:** 10.1186/s40478-022-01488-4

**Published:** 2023-01-23

**Authors:** Daniel N. El Kodsi, Jacqueline M. Tokarew, Rajib Sengupta, Nathalie A. Lengacher, Ajanta Chatterji, Angela P. Nguyen, Heather Boston, Qiubo Jiang, Carina Palmberg, Chantal Pileggi, Chet E. Holterman, Bojan Shutinoski, Juan Li, Travis K. Fehr, Matthew J. LaVoie, Rajiv R. Ratan, Gary S. Shaw, Masashi Takanashi, Nobutaka Hattori, Christopher R. Kennedy, Mary-Ellen Harper, Arne Holmgren, Julianna J. Tomlinson, Michael G. Schlossmacher

**Affiliations:** 1 Program in Neuroscience, Ottawa Hospital Research Institute, Ottawa, ON Canada; 2 Department of Cellular and Molecular Medicine, University of Ottawa, Ottawa, ON Canada; 3 Department of Biochemistry, Karolinska Institute, Stockholm, Sweden; 4 Department of Biochemistry, Microbiology and Immunology Faculty of Medicine, and Ottawa Institute of Systems Biology, University of Ottawa, Ottawa, ON Canada; 5 Kidney Research Center, Ottawa Hospital Research Institute, Ottawa, ON Canada; 6 Department of Neurology, College of Medicine, University of Florida, Gainesville, FL USA; 7 Burke Neurological Institute, Weill Cornell Medical School, White Plains, NY USA; 8 Department of Biochemistry, University of Western Ontario, London, ON Canada; 9 Department of Neurology, Juntendo University School of Medicine, Tokyo, Japan; 10 University of Ottawa Brain and Mind Research Institute, Ottawa, ON Canada; 11 Division of Neurology, Department of Medicine, The Ottawa Hospital, Ottawa, ON Canada; 12 Present Address: Snyder Institute, University of Calgary, Calgary, AB Canada; 13 Present Address: Amity Institute of Biotechnology, Amity University, Kolkata, West Bengal India

**Keywords:** Early-onset Parkinson disease, Parkin, *Prkn*, *Sod2*, Redox stress, Glutathione metabolism, Mass spectrometry, Posttranslational modification

## Abstract

We recently discovered that the expression of *PRKN*, a young-onset Parkinson disease-linked gene, confers redox homeostasis. To further examine the protective effects of parkin in an oxidative stress model, we first combined the loss of *prkn* with *Sod2* haploinsufficiency in mice. Although adult *prkn*^−/−^//*Sod2*^±^ animals did not develop dopamine cell loss in the *S. nigra*, they had more reactive oxidative species and a higher concentration of carbonylated proteins in the brain; bi-genic mice also showed a trend for more nitrotyrosinated proteins. Because these redox changes were seen in the cytosol rather than mitochondria, we next explored the thiol network in the context of *PRKN* expression. We detected a parkin deficiency-associated increase in the ratio of reduced glutathione (GSH) to oxidized glutathione (GSSG) in murine brain, *PRKN-*linked human cortex and several cell models. This shift resulted from enhanced recycling of GSSG back to GSH via upregulated glutathione reductase activity; it also correlated with altered activities of redox-sensitive enzymes in mitochondria isolated from mouse brain (e.g., aconitase-2; creatine kinase). Intriguingly, human parkin itself showed glutathione-recycling activity in vitro and in cells: For each GSSG dipeptide encountered, parkin regenerated one GSH molecule and was *S*-glutathionylated by the other (GSSG + P-SH $\to$ GSH + P-*S*-SG), including at cysteines 59, 95 and 377. Moreover, parkin’s *S*-glutathionylation was reversible by glutaredoxin activity. In summary, we found that *PRKN* gene expression contributes to the network of available thiols in the cell, including by parkin’s participation in glutathione recycling, which involves a reversible, posttranslational modification at select cysteines. Further, parkin’s impact on redox homeostasis in the cytosol can affect enzyme activities elsewhere, such as in mitochondria. We posit that antioxidant functions of parkin may explain many of its previously described, protective effects in vertebrates and invertebrates that are unrelated to E3 ligase activity.

**Supplementary Information:**

The online version contains supplementary material available at 10.1186/s40478-022-01488-4.

## Introduction

Parkinson disease (PD) is a progressive, heterogeneous disorder of the human brain that remains incurable. The *PRKN*-linked, autosomal-recessive variant of PD (ARPD) is characterized by the selective degeneration of dopamine and noradrenaline producing neurons in the *S. nigra* and *L. coeruleus,* respectively [32]. In human brain, these neurons are thought to be particularly vulnerable due to their high level of pro-oxidant radical generation during decades of normal ageing. Such redox stress stems from unique features of *S. nigra* and *L. coeruleus* neurons that include: their post-mitotic state; extensive arborization; a high number of axonal mitochondria; the relative abundance of metals in redox-reactive forms; a greater need to buffer Ca^2+^ ions; and the ongoing generation of toxic, catecholamine metabolism-linked radicals in the cytosol [5, 14, 35, 62].

Oxidative stress and mitochondrial damage have been implicated in the pathogenesis of several brain disorders including PD [41]. Mitochondrial dysfunction, as induced by neurotoxicants, such as 1-methyl-4-phenyl-1,2,3,6-tetrahydropyridine (MPTP) and rotenone, augments oxidative stress in nigral neurons [16, 28]. The integrity of the cellular thiol pool, a network formed by glutathione and the cysteine proteome, is essential in maintaining redox homeostasis in long-lived, post-mitotic cells of both the brain and heart. Depletion of the thiol pool itself is used as a measure of, and contributes to, cellular oxidative stress [20]. The reduced form of glutathione (GSH) serves a critical role as a polyvalent antioxidant. Accordingly, a decline in GSH has been implicated in many human disorders, including neurodegenerative diseases, and related pathways, thereby leading to cell death [40, 54]. Intriguingly, several reports of parkin deficiency, as modeled in murine brain and primary glial cultures, previously revealed a change in glutathione homeostasis [19, 21, 49]. The underlying mechanism, however, has remained unexplored.

We recently published that wild-type (WT), human parkin can act as a redox state-sensing and redox state-effecting protein. In this context, we found that parkin contributes to cellular homeostasis by mediating reactions that lower chronic oxidative stress. It does so in a cysteine-based, thiol-dependent manner in cell models, in murine brain and human cortex [53]. This function was found to be independent of its E3 ligase activity. When viewed in this context, it led us to ask whether parkin-dependent, redox-based mechanisms could explain heretofore unresolved findings from unbiased, proteomic-based investigations of parkin-deficient mice. Previously, Palacino et al. and Periquet et al. found that the isoelectric focusing points - but not the total abundance- of select proteins were altered in two separate models of adult, parkin-deficient murine brain [36, 38].

Based on further analysis of these two mass spectrometry-based studies, we made two observations: one, that a majority of dysregulated proteins had been previously classified as enzymes (55/92; 59.8%); and two, that many of the proteins’ activities had been described as redox-sensitive (72/92; 78.2%), e.g., glyoxalase-1 and aconitase-2 (Aco2) [36, 38]. We therefore hypothesized that parkin-dependent alterations in protein modifications, such as those conferred by redox changes, could modify activities of many enzymes throughout the cell, including those identified by Palacino et al. and Periquet et al., and thus be relevant to understanding parkin’s protective effects in the brain.

Here, we explored a possible role for *PRKN* expression in the context of redox homeostasis in vitro, in cells and in mammalian brain. We identified two parkin-dependent effects on glutathione metabolism. First, we demonstrate that parkin can directly reduce oxidized glutathione (GSSG) to GSH via its own thiols, thereby contributing to GSSG recycling, which occurs in the cytoplasm. Second, in parkin-deficiency states glutathione reductase activity was upregulated in murine and human brain, thus promoting GSSG recycling. These new findings are congruent with a protective function for parkin, which occurs at least in part, via redox chemistry-based mechanisms [11, 53].

## Materials and methods

### Mouse lines and tissues

All animal-related experiments were conducted in accordance with the Canadian Council on Animal Care Standards and the Animals for Research Act and were approved by the University of Ottawa Animal Care Council. WT C57BL/6 J mice were purchased from the Jackson Laboratory. The *prkn*^−/−^ mice (C57BL/6 J background) were obtained from Dr. A. Brice [19]. The *Sod2*^±^ mice (C57BL/6 J) were generated by Lebovitz [26], and purchased from the Jackson Laboratory; *prkn*^−/−^//*Sod2*^+/+^ and *Sod2*^±^//*prkn*^+/+^ mice were crossed to generate *prkn*^±^//*Sod2*^±^ offspring, which was interbred to produce the desired *prkn*^−/−^//*Sod2*^±^ (bi-genic) mouse. Brains and hearts were collected for the four genotypes of interest: WT; *prkn*^−/−^; *Sod2*^±^; and *prkn*^−/−^ //*Sod2*^±^.

### Genotyping

Ear tissue was collected for genotyping. DNA was extracted from the tissue by incubating the ear sample in 1× solution A (Solution A (10×): 250 mM NaOH, 2 mM EDTA, in water) at 95 °C for 30 min, followed by neutralizing the reaction with 1× solution B (Solution B (10×): 400 mM Tris-HCl, in water). Standard polymerase chain reaction (PCR) was used to amplify the *prkn* and *Sod2* loci. The following primers were used:

$$\begin{aligned} & prkn^{{ + / + }} ,{\text{ F}}{:}\;{\text{TGCTCTGGGGTTCGTC}};\;\;{\text{R}}{:}\;{\text{TCCACTGGCAGAGTAAATGT}} \\ & prkn^{{ - / - }} ,{\text{ F:}}\;{\text{TTGTTTTGCCAAGTTCTAAT}};\;\;{\text{R}}{:}\;{\text{TCCACTGGCAGAGTAAATGT}} \\ & Sod2^{{ + / + }} ,{\text{ F}}{:}\;{\text{TGAACCAGTTGTGTTGTCAGG}};\;\;{\text{R}}{:}\;{\text{TCCATCACTGGTCACTAGCC}} \\ & Sod2^{{ - / - }} ,{\text{ F}}{:}\;{\text{TGTTCTCCTCTTCCTCATCTCC}};\;\;{\text{R}}{:}\;{\text{ACCCTTTCCAAATCCTCAGC}} \\ \end{aligned}$$

Amplification products were electrophoresed on a 1% agarose gel and stained with ethidium bromide. A band at 300 base pairs (bp) represented a *prkn*^+/+^ or *prkn*^−/−^ amplification product (where a sample with 300 bp bands for both *prkn*^+/+^ and *prkn*^−/−^ primer pairs was identified as a *prkn*^±^ mouse). A band at 123 bp represented a *Sod2*^+^ genotype and a band at 240 bp represented a *Sod2*^−^ allele.

### SOD activity assay

A superoxide dismutase (SOD) assay kit (Cayman chemical) was used to measure MnSOD (SOD2) activity in mouse brain. Pre-weighed, perfused mouse brain pieces were homogenized in 5 mL cold 20 mM HEPES buffer, pH 7.2, supplemented with EGTA, mannitol and sucrose, with a Dounce homogenizer on ice. The homogenates were centrifuged at 1500×*g* 5 min at 4 °C. The resulting supernatants were centrifuged at 10,000×*g* 15 min at 4 °C to isolate the mitochondria (pellet). Two mM potassium cyanide, which inhibits Cu/Zn-SOD and extracellular SOD, was added to each sample to ensure assay specificity for MnSOD. The SOD standards were prepared by adding 200 μL of the radical detector and 10 μL of the provided standards, in duplicates in a 96-well plate. The same was repeated for the samples. The reaction was initiated by adding 20 μL of xanthine oxidase to all the wells. Background absorbance was assayed by adding 20 μL xanthine oxidase to sample buffer (optional). The plate was incubated on a shaker for 30 min at room temperature. The absorbance was measured at 450 nm. The linearized SOD standard curve was plotted and used to calculate MnSOD activity (U/mL) from averaged sample absorbance readings.

### Measurements of reactive oxygen species (ROS) in tissues

The Amplex® Red hydrogen peroxide/peroxidase assay kit (Invitrogen) was used to monitor endogenous levels of H_2_O_2_ in mouse tissues and cells. Pre-weighed hearts and specimens of cortices as well as midbrains (or pelleted cells) were homogenized on ice in the 1× reaction buffer provided, using a Dounce homogenizer (3 times volume to weight ratio). Homogenates were diluted in the same 1× reaction buffer (10× and 5×). A serial dilution of the H_2_O_2_ standard provided was prepared (20, 10, 2 and 0 μM). Fifty μL of standards and samples were plated in a 96 well black plate with clear flat bottom. The reaction was started by the addition of 50 μL working solution which consisted of 1× reaction buffer, Amplex® red and horseradish peroxidase. The plate was incubated at room temperature for 30 min protected from light. A microplate reader was used to measure either fluorescence with excitation at 560 nm and emission at 590 nm, or absorbance at 560 nm. The obtained H_2_O_2_ levels (μM) were normalized to tissue weight (in g) or protein concentration (μg/μL).

### ROS measurements in intact cells

Mammalian cells, including SH-SY5Y and HEK293 cells, were transfected with flag-tagged WT *PRKN* cDNA or control vector (pcDNA), as described before. After 24 h the cells were lifted using trypsin and re-seeded in a 12-well dish at a density of 0.3 × 10^6^ cells/mL. After 48 h the cells were treated with 0 mM or 2 mM H_2_O_2_ in OPTI-MEM medium at 37 °C and 5% CO_2_. After 1 h the cells were washed with OPTI-MEM and incubated with 20 μM of dichlorofluorescin diacetate (DCFH-DA, Sigma) for 30 min at 37 °C and 5% CO_2_. Cells were collected using a cell lifter and treated with ethidium-1 dead stain (Invitrogen) for 15 min at room temperature. Samples were analyzed using a BD Fortessa flow cytometer set to measure ROS-sensitive signals (DCFH-DA, ex. 488 nm and em. 527 nm) and viability-related stains (ethidium-1, ex, 528 nm and em. 617 nm). The results were reported as the average mean fluorescence intensity (MFI) of ROS in live cells. Each separate transfection was considered one biological replicate.

### Western blotting and densitometry

Brain and heart homogenates as well as cell lysates were run on 4–12% Bis–Tris SDS-PAGE gels using MES running buffer. Proteins were transferred to PVDF membranes using transfer buffer, and immunoblotted for parkin, DJ-1, MnSOD, Aco2, mitochondrial creatine kinase (mtCK), VDAC, TOM20, nitrotyrosine, glutathione reductase, and glyoxalase-1. Actin and Ponceau S staining were used as loading controls. For densitometry quantification, the signal intensity of protein nitrotyrosination and glutathione reductase from each sample was measured as pixel using Image J Software and controlled for total protein loading.

### Protein carbonyl assay

A protein carbonyl colorimetric assay kit (Cayman chemical) was used to assay the carbonyl content in human and mouse brains or hearts. Pre-weighed tissues were rinsed in PBS and then homogenized in 1 mL cold PBS at pH 6.7 supplemented with 1 mM EDTA, using a Dounce homogenizer on ice. Homogenates were centrifuged at 10,000×*g* for 15 min at 4 °C. Two hundred μL of the supernatant was added to a tube with 800 μL 2,4-Dinitrophenylhydrazine (DNPH; sample tube) and 200 μL of the supernatant was added to a tube with 800 μL 2.5 M HCl (control tube), both tubes were incubated in the dark for 1 h with occasional vortexing. 1 mL 20% TCA followed by 1 mL 10% TCA solutions were added to the centrifuged (10,000×*g* 10 min at 4 °C) pellet after discarding the supernatant. The resulting pellet was resuspended in 1 mL of 1:1 ethanol:ethyl acetate mixture and centrifuged 3 times to extract protein pellets. The final pellets were suspended in 500 μL guanidine hydrochloride and centrifuged. A total of 220 μL per sample and control supernatants were added to two wells of a 96-well plate, and the absorbance was measure at 360 nm. The corrected absorbance (CA, sample value minus control value) was used in the following equation to obtain the protein carbonyl concentration: Protein Carbonyl (nmol/mL) = [(CA)/(0.011 μM^−1^)](500 μL/200 μL). Total protein concentration from the sample tissues was measured to obtain the carbonyl content, i.e., protein carbonyl/total protein concentration.

### Cell cultures, transfection and oxidation

Mammalian cell cultures (CHO; HEK293; SH-SY5Y) were grown in Dulbecco’s Modified Eagle Medium (DMEM) supplemented with 1% penicillin/streptomycin and 10% heat-inactivated fetal bovine serum (FBS) at 37 °C with 5% CO_2_. For transient transfection paradigms, 4 to 15 μg of cDNA coding for *N*-terminally Flag-tagged WT human parkin or empty Flag control vector (pcDNA3.1) using a 1:1 ratio of cDNA:Lipofectamine 2000, was used for ectopic expression. The cDNA and Lipofectamine 2000 reagent were incubated for 20 min at RT before being applied to the cells for 1 h at 37 °C with 5% CO_2_, followed by direct addition of fresh culture medium. Cells were incubated another 24 h before treatment, harvesting and analysis. Chinese hamster ovary cells, stably expressing the myc-vector (CHO), or *N*-terminal myc-tagged, WT human parkin (CHO-parkin) were also used [26].

All chemicals (H_2_O_2_, DTT, BSO and NAC) were added directly to intact cells at ~ 75% confluence in growth or OPTI-MEM media. Cells were manually scraped, spun at a 100×*g* for 5 min, the pellets washed with PBS and then homogenized in a Tris salt buffer, transferred to ultracentrifuge tubes and spun at 163, 200×*g* and 4 °C for 30 min to extract the soluble fraction. The resulting pellets were further homogenized in the Tris salt buffer with the addition of 2–10% SDS, transferred to ultracentrifuge tubes and spun at 163, 200×*g* and 10 °C for 30 min to extract the insoluble fraction. SH-SY5Y cells were seeded at a density of 0.5–1 × 10^6^ cells/mL. Once cells reached 70–80% confluency they were transfected with cDNA coding for C-terminally flag-tagged parkin or empty flag control vector (pcDNA3) by electroporation using the nucleofector method described by Hu and Li, 2015. A total of 2 million cells were resuspended in 100 μL of OPTI-MEM containing cDNA (2 μg) and 1% polyoxamer 188. The cells were electroporated using the X Unit and pulse code “CA-137” on a Lonza 4D-Nucleofector. Following electroporation, cells were seeded at a concentration of 0.8–1 × 10^6^ cells/mL [18].

### Expression of recombinant, maltose-binding protein-tagged parkin

WT and truncated (amino acid residues 327–465) human parkin proteins were generated in the pMAL-2 T vector (a gift from Dr. Keiji Tanaka), as previously described [29]. Parkin produced in this vector contained an *N*-terminal maltose-binding protein (MBP) and a thrombin cleavage site (LVPRGS). All proteins were overexpressed in *E. coli* BL21 Codon-Plus competent cells (New England Biolabs) and grown at 37 °C in 2% Luria Broth containing 0.2% glucose and 100 mg/L ampicillin until OD_600_ reached 0.3–0.37, at which point protein expression was induced with addition of 0.4 mM isopropyl β-D-1-thiogalactopyranoside (IPTG). Cultures were left to express protein at 37 °C until OD_600_ reached 0.9–1.0. Harvested protein isolates were purified using amylose resin in buffers containing 100 μM zinc sulfate and 10 mM maltose.

### Expression of recombinant, tag-less parkin

WT human parkin was generated as an initially 6His-Smt3-tagged protein in *Escherichia coli* BL21 (DE3) Codon-Plus RIL competent cells (New England Biolabs), as described [3, 24, 50, 53]. Transformed bacteria were grown at 37 °C in 2% Luria Broth containing 30 mg/L kanamycin until OD_600_ reached 0.6, at which point the temperature was reduced to 16 °C. Parkin protein-expressing cultures were supplemented with 0.5 mM ZnCl_2_. Once OD_600_ reached 0.8, protein expression was induced with IPTG, except for ULP1 protease expression, which was induced once OD_600_ had reached 1.2. The concentration of IPTG used for each construct is as follows: 25 μM for WT parkin, and 0.75 mM for the ULP1 protease, as described in detail. Cultures were left to express protein for 16–20 h. Cells were harvested by centrifugation, lysed and processed via Ni–NTA agarose beads in elution columns. ULP1 was purified in a similar fashion and used to cleave the tag off of the 6His-Smt3-parkin fusion protein, thus generating full-length r-parkin (aa_1-465_), as described [11, 53].

### Cell cytotoxicity assay

A Vybrant™ cytotoxicity assay kit (Molecular Probes V-23111) was used to monitor cell death through the release of the cytosolic enzyme glucose 6-phosphate dehydrogenase (G6PD) from damaged cells into the surrounding medium. 50 μL of media alone (no cells), media from control and stressed CHO-parkin and control cells and cell lysates were added to a 96-well microplate. 50 μL of reaction mixture, containing reaction buffer, reaction mixture and resazurin, was added to all wells, and the microplate was incubated at 37 °C for 30 min. A microplate reader was used to measure resorufin fluorescence with excitation at 560 nm and emission at 590 nm. A rise in fluorescence indicates a rise in G6PD levels*, *i.e., a rise in cell death.

### Isolation of mitochondria from tissues

Freshly dissected brain tissue was cut into smaller pieces, rinsed in cold PBS, and homogenized using either a Dounce homogenizer or Waring blender in the presence of twice the tissue volume of buffer A (20 mM Hepes pH 7.4, 220 mM mannitol, 68 mM sucrose, 80 mM KCl, 0.5 mM EGTA, 2 mM Mg(Ac)_2_, 1 mM DTT, 1× protease inhibitor (Roche)). The sample was centrifuged at 4070×*g* in a tabletop centrifuge for 20 min at 4 °C. The supernatant was collected and spun again as above. The resulting supernatant was spun at 10,000×*g* for 20 min at 4 °C and the pellet was then washed in the above buffer and spun again at 10,000×*g* for 20 min. The resulting mitochondrial pellet was resuspended in buffer B (20 mM Hepes pH 7.4, 220 mM mannitol, 68 mM sucrose, 80 mM KCl, 0.5 mM EGTA, 2 mM Mg(Ac)_2_, 10% glycerol), aliquoted, and snap frozen.

### Aconitase assay

The Aconitase Enzyme Activity Microplate Assay Kit (MitoSciences) was used to measure activity in mitochondria isolated from mouse brain as per manufacturer’s instructions. Two brains each from 12 month-old mice were pooled to provide enough mitochondria and normalized for total protein concentration. These were treated with 0 or 4 µM H_2_O_2_ just prior to assay. The catalytic conversion of isocitrate to cis-aconitate by aconitase was measured by quantifying the amount of cis-aconitate in the reaction by reading the samples at 240 nm. Rates in µM/min were determined from 3 independent experiments performed in triplicate.

### Creatine kinase assay

The EnzyChrom Creatine Kinase Assay Kit (BioAssay Systems) was applied to measure activity in mouse brain mitochondria (as above). Two brains each from 12 month-old mice were pooled to provide enough mitochondria and normalized for total protein concentration. Mitochondria were incubated with 0 or 0.5 mM H_2_O_2_ at room temperature for 20 min prior to assay start. The creatine kinase-dependent catalytic conversion of creatine phosphate and ADP to creatine and ATP was quantified indirectly by measuring NADPH at 340 nm. ATP produced by the reaction phosphorylates glucose to glucose-6-phosphate (G6P) by hexokinase, which is oxidized by NADP^+^ in the presence of G6P-dehydrogenase, yielding NADPH. Rates in uM/min were calculated for 3 independent experiments done in triplicate.

### Glutathione quantification by HPLC

Human and mouse brain specimens as well as pelleted CHO cells were homogenized in buffer containing 125 mM sucrose, 5 mM TRIS, 1.5 mM EDTA, 0.5% trifluoroacetic acid (TFA) and 0.5% mycophenolic acid (MPA) in mobile phase. Samples were spun at 14,000×*g* at 4 °C for 20 min. Supernatants were collected and analyzed using an Agilent HPLC system equipped with a Pursuit C_18_ column (150 × 4.6 mm, 5 µm; Agilent Technologies) operating at a flow rate of 1 mL/min. The mobile phase consisted of 0.09% TFA diluted in ddH2O and mixed with HPLC-grade methanol in a 90:10 ratio. Standard solutions were used to estimate the retention times for GSH and GSSG. Using Agilent Chemstation software, the absolute amounts of GSH and GSSG were calculated by integrating the area under the corresponding peaks, and values were calculated from standard curves.

### Glutathione concentration determined by monochlorobimane assay

Stock solutions of assay dye (monochlorobimane (MCB), 22 mM) and glutathione-*S*-transferase (50 units/mL) were prepared in PBS and stored protected from light at − 20 °C. The working solution was prepared using 12.8 μL of stock MCB and 80 μL of stock glutathione-*S*-transferase in 4 mL PBS and stored on ice. Samples were prepared as follows: cells were lifted mechanically using cell-lifters, washed twice and re-suspended in ice-cold PBS, mixed by vortex and incubated on ice for 30 min. Following two freeze thaw cycles using solid CO_2_, the samples were sonicated 1 min on wet ice (S220 Ultra-sonicator from Covaris) and spun at 3000×*g*, 4 °C, for 5 min. Total protein concentration of supernatants was determined using Bradford assay. Samples and glutathione (GSH) standards (0–13 μM) were plated in 25 μL aliquots in a 96-well plate with clear bottom and black sides. Twenty-five μL of working solution was added to all experimental wells and protected from light for 15 min at room temperature. Fluorescence (ex 380 nm, em 461 nm) was measured using a Synergy H1Multi-Mode Plate Reader (Bio Tek). The amount of GSH detected in each sample was calculated using the regression curve obtained from the glutathione standards.

### Tietze’s enzymatic recycling method to quantify glutathione

The enzymatic recycling method described by Rahman et al. [39] was used to determine GSH and GSSG levels in mouse brain lysates. Hemibrains of WT (n = 3) and *prkn* knock-out (n = 3) mice, at ~ 12 months of age respectively, were collected, weighed and homogenized in 3× v/w of KPE-X (0.1 M potassium phosphate, 5 mM EDTA, 0.1% Triton X-100, 0.6% sulfosalicylic acid, pH 7.5) using a glass Dounce homogenizer (50 passes). Samples were spun at 8000×*g* at 4 °C, for 5 min and the supernatant protein concentration was determined using a Bradford assay. To determine the total glutathione (GSH + 2GSSG) concentration, the following stock solutions were freshly prepared in KPE (0.1 M potassium phosphate, 5 mM EDTA, pH 7.5): 5,5ʹ-dithio-*bis*-2-nitrobenzoic acid (DNTB) at 0.6 mg/mL, NADPH at 0.6 mg/mL and glutathione reductase at 3 units/mL. GSH standards were prepared in KPE at concentrations of 0–26 nM/mL. Twenty μL of diluted sample or GSH standard was added per well and 120 μL of a 1:1 mixture of the DNTB and glutathione reductase stocks solutions was added to each assayed well. After 30 s incubation, 60 μL of the NADPH was added and absorbance was immediately measured at 412 nm in 30 s intervals for a total of 2 min.

To determine the level of GSSG, samples were diluted (1:4) in KPE and treated with 0.2% 2-vinylpyridine for 1 h at RT. Excess vinyl-pyridine was quenched with 1% triethanolamine and GSSG was measured using the same method as total glutathione except for: GSH standards were replaced with GSSG (0 to 26.24 nM/mL) treated with vinyl-pyridine and triethanolamine. Absolute values for total glutathione (GSH + 2GSSG) and GSSG per sample were calculated using the linear regression obtained from the change in absorbance/min plotted against GSH or GSSG standards, respectively, divided by the total protein concentration. Absolute values for GSH were determined using the equation: GSH = [GSH + GSSG] − 2 [GSSG] [39].

### Total RNA isolation, cDNA synthesis and PCR-based amplification

Pre-weighed, murine cortices were homogenized in QIAzol (Qiagen), at 1 mL volume per 100 mg of tissue, and incubated at room temperature for 5 min. 0.2 mL of chloroform per 1 mL QIAzol was added and the homogenates were shaken vigorously for 15 s, followed by a 2–3 min incubation at RT, the tubes were centrifuged at 12,000×*g* for 15 min at 4 °C. The upper clear aqueous layer was transferred to a new tube and 1 volume of 70% ethanol was added and mixed by vortexing. The solution was then added to an RNAeasy Mini spin column (Qiagen) placed in a 2 mL collection tube and centrifuged for 15 s at 8000×*g* at RT. The flow-through was discarded and 700 μL Buffer RW1 was added to the spin column and spun for 15 s at 8000×*g*. The same step was repeated with 500 μL Buffer RPE, one spin for 15 s and a second spin for 2 min. An optional spin in a new collection tube at full speed for 1 min to remove excess buffer was added. The RNAeasy Mini spin column was placed in a new collection tube, 50 μL RNase-free water was added directly to the membrane and centrifuged for 1 min at 8000×*g*. A NanoDrop machine was used to measure the amount of total RNA obtained from the cortices. Turbo DNA-free™ (Life Technologies) was used to remove trace to moderate amounts of contaminating DNA. SuperScripT™ IV First-Strand Synthesis System (Invitrogen) was used for cDNA synthesis reaction. iTaq™ Universal SYBR® Green Supermix (BIO-RAD) and select primer sets were used for PCR amplification of the newly synthesized cDNA templates, and controls, and analyzed by agarose gel electrophoresis and ethidium bromide staining. The following primers were used [9, 44]:

$$\begin{aligned} & {{DJ}} {-} 1,\;{\text{F}}{:}\;{\text{ATCTGAGTCGCCTATGGTGAAG}};\;\;{\text{R}}{:}\;{\text{ACCTACTTCGTGAGCCAACAG}} \\ & {{GCLC}},\;{\text{F}}{:}\;{\text{ATGTGGACACCCGATGCAGTATT}};\;\;{\text{R}}{:}\;{\text{TGTCTTGCTTGTAGTCAGGATGGTTT}} \\ & {{GCLM}},\;{\text{F}}{:}\;{\text{GCCACCAGATTTGACTGCCTTT}};\;\;{\text{R}}{:}\;{\text{CAGGGATGCTTTCTTGAAGAGCTT}} \\ & {{Actin}},\;{\text{F}}{:}\;{\text{CTTCCTCCCTGGAGAAGAGC}};\;\;{\text{R}}{:}\;{\text{AAGGAAGGCTGGAAAAGAGC}} \\ \end{aligned}$$

### Glutathione reductase activity assay

A glutathione reductase (GR) enzyme assay kit (Cayman Chemical) was used to measure its activity in tissues. Human and mouse brains were either perfused or rinsed with PBS (pH 7.4). Tissues were homogenized in 5–10 mL of cold buffer (50 mM potassium phosphate, pH 7.5, 1 mM EDTA) per gram of tissue, followed by centrifugation at 10,000×*g* for 15 min at 4 °C. In a 96 well clear plates, three wells were loaded with 120 μL assay buffer (provided) and 20 μL GSSG (provided) as background control; and three wells were loaded with 100 μL assay buffer, 20 μL GSSG and 20 μL diluted GR (provided) as positive control. Twenty μL of samples supernatant were loaded in triplicates, with 100 μL assay buffer and 20 μL GSSG. Reactions were initiated by adding 50 μL NADPH (provided) to each well. The 96-well plate was gently shaken for a few seconds and absorbance was read at 340 nm once every minute for 10 min, or to obtain readings at a minimum of 5 time points. The oxidation of NADPH to NADP^+^ is accompanied by a decrease in absorbance at 340 nm and is directly proportional to the GR activity in the sample.

### Parkin-mediated redox recycling of glutathione

Parkin protein buffer exchange to T200 protein buffer (50 mM Tris, 200 mM NaCl, pH 7.5) was first performed using repeat centrifugations (8 times 4000×*g* at 4 °C for 10 min) in Amicon Ultra 10 kDa MWCO filters. Protein concentration was adjusted to 10 μM using T200. Both GSH and GSSG stocks were prepared in PBS at concentrations of 1 mg/mL (3250 μM) and 2.01 mg/mL (6560 μM) respectively. Glutathione standards of 0, 2.5, 5, 10 μM and 100 μM of both GSH and GSSG were prepared and combined in the following ratios to a final volume of 90 μL: 10 μM GSH: 0 μM GSSG, 9 μM GSH: 1 μM GSSG, 8 μM GSH: 2 μM GSSG, 6 μM GSH: 4 μM GSSG, 4 μM GSH: 6 μM GSSG, 2 μM GSH: 8 μM GSSG, 1 μM GSH: 9 μM GSSG, and 0 μM GSH: 10 μM GSSG. Full-length r-parkin (1 μL of a 10 μM solution) was added to the prepared mixtures and allowed to incubate at room temperature for 15 min. Samples were analyzed for GSH concentration using the monochlorobimane assay described above.

### Glutathionylation assays

*S-*glutathionylation of recombinant parkin proteins was performed, as described previously [6]. MBP-tagged parkin proteins were eluted from columns with excess maltose. Concentrated eluates were supplemented with 0.1% DMSO (10 µL DMSO in 10 mL PBS), and excess DTT and maltose were removed by several cycles of centrifugation with 30 kDa cut-off filters. Proteins/peptides (at 14 µM) were incubated with 3 mM GSH for 1 h and then with 5 mM GSSG for 2 h at room temperature. Trypsin digestion was performed (Peptide:Trypsin = 20:1) overnight at 4 °C. Trypsin-digested fragments were run through MALDI analysis. To monitor *S*-glutathionylation, eosin-labeled GSSG (Di-E-GSSG) was used to glutathionylate proteins, as described [43]. Di-E-GSSG has quenched fluorescence in the disulphide form. Fluorescence increases ~ 20-fold upon reduction of its disulphide bond following the formation of E-GSH. Blackened 96-well-plates were used in a PerkinElmer Victor3 multilabel counter containing a final well volume of 200 μL in 0.1 M potassium phosphate buffer (pH 7.5), 1 mM EDTA. The reaction was started by addition of 20 μM Di-E-GSSG to parkin proteins, followed by recording the fluorescence emission at 545 nm after excitation at 520 nm. Controls with no peptide added were used as fluorescent background.

To confirm *S*-glutathionylation, reaction products were tested for possible deglutathionylation. Aliquots of *S*-glutathionylated proteins were treated with 10 mM DTT or with the complete GSH-glutaredoxin (Grx) system. All samples were run on a non-reducing SDS-PAGE, containing 4–12% acrylamide. The gel was exposed to UV transilluminator to visualize eosin-tagged glutathionylated protein. The same gels were later stained with Coomassie Blue dye. Di-Eosin-GSSG was purchased from IMCO or Cayman Chemical (11,547), Sweden. Human Grx-1, and Grx-2 were prepared, as described [43]. Rat recombinant thioredoxin was a kind gift from Prof. Elias Arner.

### Detection of *S-*glutathionylated parkin in cultured cells

*S*-glutathionylation of parkin in cells was performed using a modified BioGEE protocol described by Sullivan et al. [51]. CHO cells stably expressing myc-parkin [25] were treated with or without 20 µM BioGEE (Invitrogen G36000) for 3 h with the addition of 1 mM H_2_O_2_ to the media for the final 10 min. Cells were collected, pelleted and washed once with ice-cold 1× PBS, followed by two washes with 1× PBS + 50 mM iodoacetamine (IAA) (BioRad) with 5 min of rocking at RT for the second wash. The cells were then lysed in 200 µL 1× RIPA buffer + 50 mM IAA and incubated on ice for 30 min followed by a snap freeze. Lysates were thawed on ice, sonicated twice for 20 s, centrifuged at 14,000×*g* for 10 min at 4 °C, and protein concentration was equalized. The lysates were pre-cleared by incubation with biotin-blocked streptavidin-conjugated magnetic beads. For this, 50 µL of beads (Pierce) were blocked with free D-Biotin (Novabiochem) (3 mg/mL prepared in alkaline water (pH 10.5)) and rocked for 1 h at RT, followed by 5 washes with 1× PBS and incubation with the cell lysates (50 µL) for 30 min at 4 °C with rocking to remove non-specific binding of proteins to the bead complex. The pre-cleared lysate was then incubated with 50 µL of pre-washed (unblocked) streptavidin-conjugated beads and rocked for 1 h at 4 °C. The beads were washed 5 times with 10× volume of ice-cold RIPA buffer, twice with 10× volume of 0.1% SDS in PBS and then resuspended in 1 volume of 0.1% SDS/PBS and incubated for 30 min at RT. Supernatant from this incubation was saved and labelled “-DTT eluate”. Beads were then resuspended in 1 volume of 0.1% SDS in PBS containing 10 mM DTT, and rocked for 30 min at RT. The supernatant was saved and labelled “ + DTT eluate”. The eluates were passed through a 10 kDA cut-off filter and concentrated by centrifugation at 14,000×*g* for 20 min at 4 °C; the retentate was collected by centrifugation at 1000×*g* for 5 min at 4 °C. Samples were then resolved by SDS-PAGE using a 10% gel under reducing conditions. Parkin was detected by Western blot analysis using a polyclonal anti-parkin antibody (Cell Signaling, 2132S).

### Mass spectrometry analysis of *S-*glutathionylated parkin

*S-*glutathionylated proteins were treated with trypsin and the resulting peptides were separated using one dimension of liquid chromatography (LC). The LC eluent was interfaced to a mass spectrometer using electrospray ionization and peptides were analyzed by MS. LC–MS/MS analyses were performed using an Easy-nLC chromatography system directly coupled online to a Thermo Scientific Q Exactive hybrid quadrupole-Orbitrap mass spectrometer with a Thermo Scientific™ Nanospray Flex™ ion source. The sample was injected from a cooled autosampler onto a 10 cm long fused silica tip column (SilicaTips, New Objective, USA) packed in-house with 1.9 μm C18-AQ ReproSil-Pur (Dr. Maisch, Germany). The chromatographic separation was achieved using an acetonitrile (ACN)/water solvent system containing 0.1% formic acid and a gradient of 60 min from 5 to 35% of ACN. The flow rate during the gradient was 300 nL/min. MS/MS data were extracted and searched against in-house Mascot Server (Revision 2.5.0), a search engine that uses mass spectrometry data to identify and characterize proteins from sequence databases. The following parameters were used: trypsin digestion with a maximum of two missed cleavages; Carbamidomethyl (–C), Oxidation (–M), Deamidated (–NQ) and Glutathione (–SG) as variable modifications; and a precursor mass tolerance of 10 ppm and a fragment mass tolerance of 0.02 Da. The identified protein was filtered using 1% false discovery rate and at least two peptides per protein as limiting parameters.

### Statistical analyses

All statistical analyses were performed using GraphPad Prism version 8 (GraphPad Software www.graphpad.com). Differences between two groups were assessed using a Student’s t-test. Differences among 3 or more groups were assessed using a 1-way or 2-way ANOVA followed by Tukey or Dunnett post hoc corrections (as indicated) to identify statistical significance. Subsequent post hoc tests are depicted graphically and show significance between treatments. For all statistical analyses, a cut-off for significance was set at 0.05. Data are displayed with p values represented as *p < 0.05, **p < 0.01, ***p < 0.001, and ****p < 0.0001.

## Results

### Generation of a bi-genic, ***prkn***^−/−^//***Sod2***^±^-mutant mouse model

**Fig. 1:**
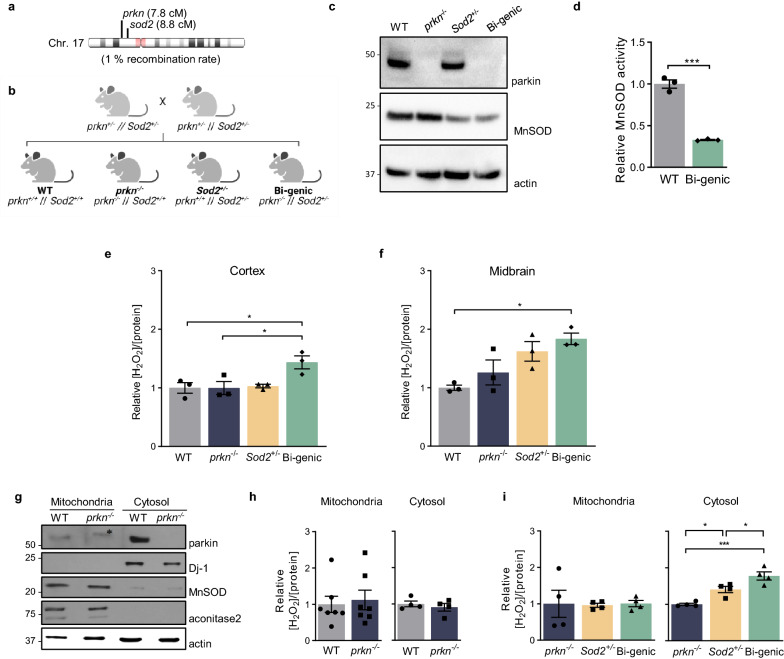
Parkin deficiency increases cytosolic hydrogen peroxide in the brain when MnSOD activity is reduced. **a** Schema of mouse chromosome 17, where *prkn* and *Sod2* loci are separated by 1 centimorgan (cM) and **b** the breeding strategy used to generate bi-genic *prkn*^*−/−*^*//Sod2*^±^ mice and littermate controls. **c** Representative Western blot of parkin, MnSOD and actin levels from ~ 3 month-old mouse brains (representative of n = 3 mice/genotype). **d** Relative MnSOD activity in isolated mitochondria from whole brain lysates of wild-type (WT) and bi-genic littermates, as shown in (c). **e–f** Ratio of endogenous levels of H_2_O_2_ (μM) to total protein concentration (μg/μL) in the cortex (**e**) and midbrain (**f**) homogenates from 6 month-old mice. **g** Representative Western blots of constituents from mitochondrial and cytosolic fractions of WT and *prkn*^*−/−*^ mouse brains, with parkin, Dj-1, MnSOD, aconitase-2 and actin as markers (*denotes a non-specific band). **h**–**i** Ratio of endogenous levels of H_2_O_2_ (μM) to total protein concentration (μg/μL) in mitochondria-enriched (**h**) and cytosolic fractions of the brain (**i**) from 6 to 8 month-old WT and *prkn*^*−/−*^ animals (left panel), and from 2 to 4 month-old *prkn*^*−/−*^*, Sod2*^±^ as well as bi-genic mice (right panel). Data represent the mean normalized to WT using n = 3/genotype **d**–**f** or n = 4–7/genotype (**h**–**i**) ± SEM. Significance was determined using unpaired Student T-test **d, h** and 1-way ANOVA with Tukey’s post-hoc (**e**, **f**, **i**), where *p ≤ 0.05, **p ≤ 0.01, and ***p ≤ 0.001

We previously showed that parkin can lower oxidative stress through a thiol-based redox mechanism [53]. Therefore, and because genomic *prkn* deficiency alone does not cause degeneration of *S. nigra* neurons in mice, we sought to further dissect redox-based mechanisms by which parkin contributes to antioxidant effects in vivo. We first crossed *prkn*^−/−^ animals [19] with *Sod2*^±^ mice [26] to test the hypothesis that parkin can modulate oxidative stress originating from mitochondrial dysfunction, such as due to the loss of a *Sod2* allele*. Sod2* encodes manganese superoxide dismutase (MnSOD), an enzyme that is the first line of defense against the rise of reactive oxygen species (ROS) within mitochondria. Mice were maintained on a C57BL/6 J background. Offspring heterozygous at both the *prkn* and *Sod2* loci were interbred over 10 generations to allow for a recombination event, which occurred at a ~ 1% rate [10], to generate the *prkn*^−/−^//*Sod2*^±^ genotype (referred to herein as bi-genic) and littermate controls (Fig. 1a, b). The bi-genic mouse does not express detectable parkin protein and has reduced MnSOD protein as well as activity levels, when compared to *Sod2*^+*/*+^ littermates (Fig. 1c, d). Quantification of superoxide anion (O_2_^−^) levels in the brain using HPLC also confirmed significantly altered levels in the bi-genic and *Sod2*^±^ mice versus their littermates (not shown).

### ***Prkn***^−/−^//***Sod2***^±^ mice show elevated ROS levels in the brain

Brains of WT, *prkn*^−/−^, *Sod2*^±^, and bi-genic (*prkn*^−/−^//*Sod2*^±^) animals were analyzed at 6 months of age. Parkin deficiency alone was insufficient to generate a significant rise in H_2_O_2_ concentrations in adult mouse brain under basal conditions (Fig. 1e, f and [53]). When coupled to haploinsufficiency at the *Sod2* locus, however, there was a significant increase in endogenous ROS concentrations in the cortex and midbrain of bi-genic mice compared to littermates (Fig. 1e, f). Unexpectedly, this rise in H_2_O_2_ levels occurred in the cytosol and was not observed in isolated mitochondria, as seen in *Sod2*^±^ mutant and bi-genic animals (but not in *prkn*^*−/−*^mice, Fig. 1g, h, i), as early as 2–4 months of age (*p* < 0.05 and *p* < 0.001, respectively; Fig. 1i, right panel).

### Parkin expression lowers chronic oxidative stress in the brain

**Fig. 2:**
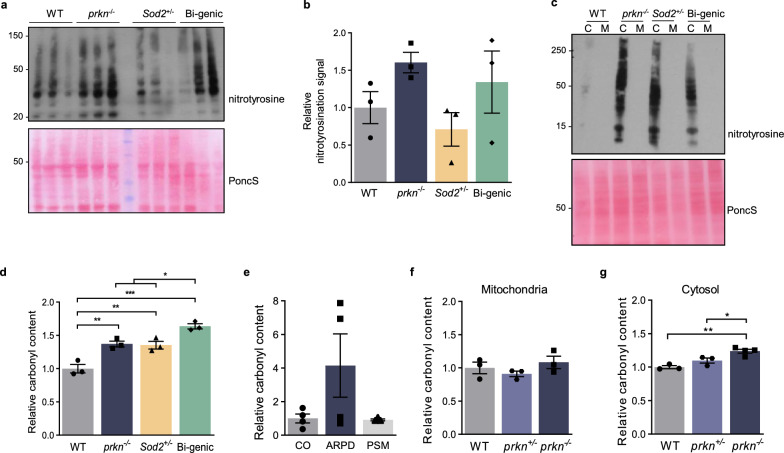
Parkin lowers chronic oxidative stress-induced damage in the cytosol of mammalian brain. **a**, **b** Total protein nitrotyrosination in midbrain homogenates from 6 month-old mice of indicated genotypes, where each lane represents a separate mouse. Ponceau S was used as a loading control to quantify relative nitrotryrosine signals (**b**). **c** Total protein nitrotyrosination in cytoplasmic (C) versus mitochondrial (M) fractions from midbrains of 6 month-old mice. **d** Relative, mean protein carbonyl content in brain homogenates from 6 month-old mice, and **e** homogenates of human frontal cortices from control subjects and age- and ethnicity-matched, parkin-deficient autosomal recessive Parkinson disease (ARPD) patients as well as from age-matched, non-*PRKN-*linked parkinsonism (PSM) cases. **f** Relative, mean protein carbonyl content of brain mitochondria and in **g** of cytosolic fractions from 6-month-old WT, *prkn*^±^ and *prkn*^*−/−*^ mice. Data in **b**–**g** represent n = 3–4/genotype ± SEM. Significance was determined using a 1-way ANOVA with Tukey’s post-hoc analysis (**b, d**–**g**), where *p ≤ 0.05, **p ≤ 0.01, and ***p ≤ 0.001

We next probed the same tissues for evidence of irreversible damage incurred by reactive nitrogen species (RNS). Protein nitrotyrosination occurs downstream of rising superoxide levels that result in sustained, RNS-mediated stress. Quantification of the peroxynitrite-linked signals of immunoblots of midbrain lysates from *prkn*^−/−^ mice showed a trend toward higher levels of protein nitrotyrosination compared to WT littermates, as did bi-genic animals (Fig. 2a, b). Similar results for ROS and nitrotyrosination levels were observed in homogenized hearts from the same mice (Additional file 1: Fig. S1a–c). Consistent with our findings of a cytosolic increase in ROS concentrations, elevated protein nitrotyrosination was generally detectable in the cytoplasm but not in mitochondria-enriched fractions, as shown in midbrain homogenates (Fig. 2c).

We next quantified protein carbonylation in tissue lysates from the 6 month-old mice. Protein carbonylation is an irreversible, posttranslational modification caused by chronic ROS-mediated stress. Carbonyl content was significantly increased in the cytosol of *prkn-*deficient brain, even under basal conditions, consistent with a previous report by Palacino et al. [36] (Fig. 2d). The carbonyl content was also increased in the *Sod2*^±^ mice; it was further elevated in the bi-genic mice (*p* < 0.01; Fig. 2d), thus demonstrating a protective role of *prkn* expression. Notably, in the absence of parkin, protein carbonyl levels showed a trend toward elevation in human cortices from mutant *PRKN*-linked ARPD versus control subjects; the latter had been matched for age, *post mortem* interval and ethnicity (Fig. 2e) [46]. Of note, the same ARPD cortices showed significantly elevated H_2_O_2_ concentrations [53]. In contrast, non-*PRKN-*linked parkinsonism cases revealed the same degree of carbonyl content as age-matched controls (Fig. 2e). Finally, in mouse brain we observed a significant *prkn-*allele dosage effect on protein carbonylation in the cytosol, but not in isolated brain mitochondria (*p* < 0.05; Fig. 2f, g). Together, these results demonstrated that endogenous, murine parkin expression was correlated with lower levels of chronic, ROS-dependent (and possibly, RNS-linked) oxidative damages in the brain.

Nevertheless, these biochemical changes in mice were insufficient to cause the death of dopamine neurons in the *S. nigra pars compacta* by 1 year of age, as assessed by stereological quantification of tyrosine hydroxylase-positive cells across the four genotypes [11], which matched the findings of Hennis et al. in a similar model [17]. Therefore, we sought to explore compensatory mechanisms that underlie the resilience of adult *prkn*^−/−^ mice to buffer chronically augmented, oxidative stress. We hypothesized that clues may lie in the cytosol-based regulation of the thiol network by parkin, likely mediated by redox-reactive cysteines [53].

### Parkin contributes to the thiol network during oxidative stress

Regulation of the pool of thiols, which includes glutathione, is an essential antioxidant mechanism in eukaryotic cells [20]. Therefore, we first examined the survival of stably transfected CHO-parkin cells following exposure to H_2_O_2_ and after depletion of GSH with a γ-glutamyl-cysteine synthetase inhibitor, l-buthionine-sulfoximine (BSO). Of note, we chose CHO cells due to their relative resilience toward redox stressors [59]. Treatment with either H_2_O_2_ or BSO alone did not cause significant cell death compared to control conditions (Additional file 1: Fig. S2a). However, combining the two stressors led to a significant parkin-dependent decrease in ROS levels (*p* < 0.001) and cell death (*p* < 0.05) (Additional file 1: Fig. S2b, c). As expected, treatment with excess *N*-acetylcysteine, an exogenous source of reactive thiols, was also protective and masked parkin-dependent outcomes (Additional file 1: Fig. S2b, S2c). Together, these findings suggested a protective mechanism by which parkin contributed to the network of available thiols during oxidative stress. We next explored the effects of exogenous WT parkin expression on the metabolism of glutathione.

### *PRKN* cDNA expression changes glutathione metabolism in cells

We first measured the levels of cellular GSH and GSSG (oxidized glutathione) by HPLC. We found that CHO cells that stably overexpress myc-parkin (CHO-parkin) [25] had significantly decreased concentrations of GSH, increased concentrations of GSSG and a reduced GSH:GSSG ratio compared to stably transfected vector-control CHO cells, but without any detectable change in the total concentrations of GSH and GSSG (Additional file 1: Fig. S2d). The same parkin-dependent changes in GSH levels and the GSH:GSSG ratio were observed in transiently transfected, human dopaminergic SH-SY5Y cells (*p* < 0.01; Additional file 1: Fig. S2e) and HEK293 cells (not shown). Consistent with the reported, high stress tolerance of CHO cells [59], adding exogenous H_2_O_2_ did not further change the relative GSH concentrations (or the GSH:GSSG ratio) between CHO-parkin versus CHO-control cells; however, exposure to ROS-mediated stress now lowered relative GSSG concentrations in the presence of excess parkin and led to a rise in total glutathione levels in its absence (Additional file 1: Fig. S2f).

### Parkin participates in glutathione recycling

**Fig. 3:**
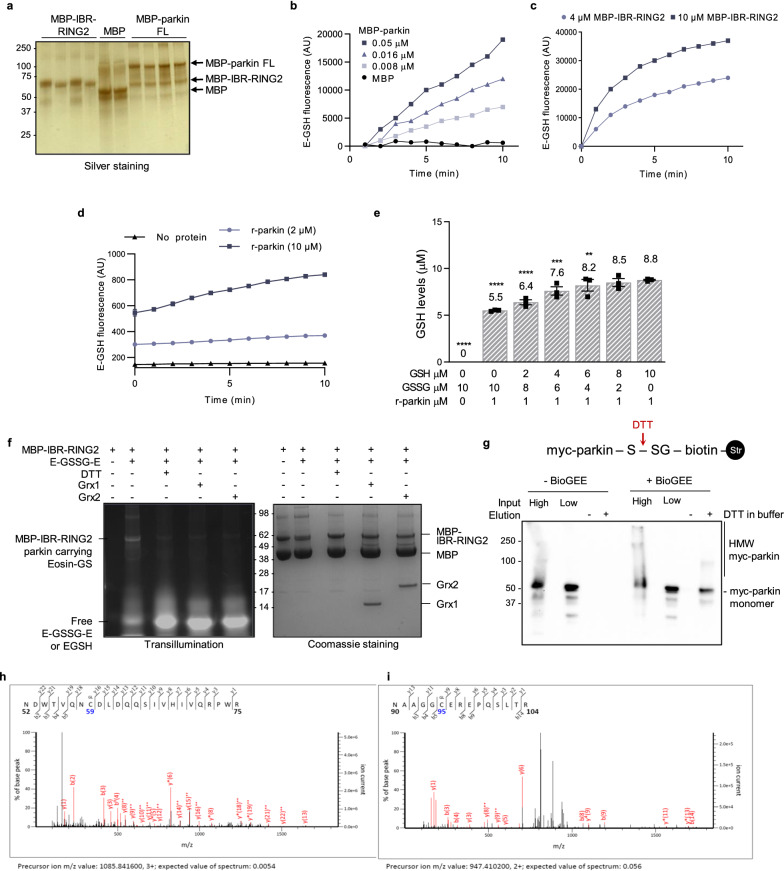
Parkin mediates the recycling of oxidized to reduced glutathione, resulting in its own *S-*glutathionylation. **a** Silver staining of recombinantly expressed maltose binding protein (MBP; tag only) and MBP-tagged, human parkin proteins separated on SDS/PAGE under reducing conditions. **b**–**d** Fluorescence-based quantification of eosin (E)-labelled GSH following incubation of full-length (FL) MBP-parkin (**b**), MBP-IBR-RING2-parkin (**c**), or untagged, recombinant (r-) human parkin (**d**) with 20 mM Di-E-GSSG, monitored over 10 min; (n = 2 runs (a, b, c) in triplicate wells). **e** Quantification of free GSH levels, as measured in the monochlorobimane assay, following incubation of indicated levels of untagged glutathione (mM) at various GSH:GSSG ratios in the presence of 1 mM of untagged, full-length, human r-parkin (n = 3 ± SEM). A 1-way ANOVA with Dunnett’s post-hoc test was used to compare all values to r-parkin incubated with 10 μM GSH, where *p ≤ 0.05, **p ≤ 0.01, and ***p ≤ 0.001. **f** In vitro* S-*glutathionylation studies of recombinant parkin, where preparations of 10 mM MBP-IBR-RING2 parkin were treated with 20 mM Di-E-GSSG followed by SDS/PAGE (lanes 1 and 2; both panels). Deglutathionylation studies in the presence of 5 mM DTT alone or in the presence of either 1 mM glutaredoxin 1 (Grx1) or Grx2 (together with: 1 mM NADPH; 5 mM GSH; 0.1 mM glutathione reductase), as indicated (lanes 3–5). Left panel shows a transilluminated gel; the right panel a Coomassie-stained gel. Results are representative of three independent experiments. **g** (upper) Schema of streptavidin-based enrichment of cellular biotin-labelled *S*-glutathionylated myc-parkin following treatment of cells with biotin-tagged GSSG (BioGEE) where *S*-glutathionylated proteins elute from the streptavidin beads in the presence of DTT and are then resolved by SDS-PAGE. (lower) Western blot of *S*-glutathionylated myc-parkin isolated from CHO-parkin cells either untreated (−) or treated with (+) BioGEE (20 µM; 3 h). Both were exposed to 1 mM H_2_O_2_ for 10 min prior to lysis. A fraction of input lysate is shown for each condition; high (5 µg) and low (2 µg). **h,i** Examples of LC–MS/MS-generated spectra following trypsin digestion of MBP-parkin proteins incubated with Di-E-GSSG showing *S*-glutathionylation (as in f) are shown at two residues: (**h**) identification of residue Cys59 within human parkin peptide aa 52–75, and in (**i**) of Cys95 within human parkin peptide aa 90–104

The observed *PRKN* overexpression-dependent lowering in relative GSSG concentrations during oxidative stress led us to explore whether parkin has a direct effect on glutathione metabolism, such as through an interaction with GSSG, as informed by its interactions with select oxidants, e.g., H_2_O_2_ and dopamine radicals [53]. We first tested this in vitro using highly purified, recombinant proteins (Fig. 3a) and eosin-labelled GSSG (i.e., E-GSSG-E, referred to as Di-E-GSSG), which by itself does not emit fluorescence (Fig. 3b). There, full-length, MBP-tagged human parkin, but not MBP alone, had concentration-dependent activity in reducing the Di-E-GSSG probe to E-GSH, measured by a rise in fluorescence at 545 nm [43] (Fig. 3b). *N*-terminally truncated parkin comprising the IBR-RING2 domains (aa 327–465) and a C-terminal RING2 peptide (aa 413–465) also showed E-GSH-regenerating activity (Fig. 3c; and data not shown). In these experiments the reactions by MBP-parkin proteins were carried out in the presence of 1 mM EDTA that competes with parkin’s chelation of divalent ions, thereby initiating its unfolding [53]. These E-GSH-regenerating findings were confirmed using untagged, human WT parkin (r-parkin) [53], as prepared in the absence of EDTA (Fig. 3d), and as conducted in an independent laboratory.

We then tested whether the reduction of GSSG to GSH in this assay was due to a direct interaction of parkin with GSSG (Fig. 3e–g). First, we incubated r-parkin (1 μM; in the absence of EDTA) with untagged, eosin-free preparations of variable concentrations of GSH and GSSG molecules at the total concentration of 10 μM per reaction in each well (Fig. 3e); thereafter, we specifically measured the net concentration of reduced GSH. We determined that under these conditions, human parkin was able to reduce the equivalent of one GSH molecule for every GSSG dipeptide (Fig. 3e). These findings revealed a direct redox effect by WT human parkin onto its surrounding thiol network in vitro.

### *S*-glutathionylation of recombinant parkin and its reversal by glutaredoxin

The stoichiometry of the observed reactions suggested that for every recycled molecule of GSSG in vitro parkin itself could be *S*-glutathionylated. Indeed, we confirmed the formation of *S-*glutathionylated parkin (referred to as parkin-*S*-SG-E), following incubation of MBP-parkin with the Di-E-GSSG probe, using SDS-PAGE of the reaction products, and as performed under non-reducing conditions. There, we visualized parkin proteins conjugated by eosin-tagged -SG moieties using UV light, as shown for example for MBP-IBR-RING2-parkin (Fig. 3f, lane 2 of left panel). This reaction was dependent on the oxidized Di-E-GSSG probe being present.

In the same experiment, we found that the *S*-glutathionylation of MBP-IBR-RING2-parkin was reversible by activated glutaredoxin-1 and -2 [43] (Fig. 3f; lanes 4 and 5 of both panels) as well as DTT (as a positive control; Fig. 3f; lane 3 in left panel), but not by thioredoxin-1 (as a negative control; not shown) [48]. Taken together, these results suggested that parkin’s ability to interact with GSSG dipeptides in vitro raised the net concentration of GSH, which in the process led to *S*-glutathionylation of parkin itself (GSSG + P-SH $\to$ GSH + P-*S*-SG); the latter modification was specifically reversible by glutaredoxin-1 and -2.

### *S-*glutathionylation of parkin occurs in living cells

The *S*-glutathionylation of human parkin was next examined in living cells using a biotinylated glutathione probe (BioGEE) under oxidative stress conditions, as published [27, 51] (Fig. 3g, schematic). We confirmed the generation of parkin-*S*-SG in BioGEE-treated CHO-parkin cells (~ 53 kDa; Fig. 3g); the extent of parkin oxidation under these conditions was readily apparent in the formation of high molecular weight smears in cell lysates prior to pulling down *S*-glutathionylated proteins (Fig. 3g; input lanes) [25, 53].

### *S-*glutathionylation of parkin occurs at several cysteine residues

The *S*-glutathionylation of parkin was further confirmed by LC–MS/MS and MALDI analysis of trypsin-digested, human MBP-parkin-*S*-SG-E preparations under non-reducing conditions. There, we mapped *S*-linked glutathionylation modifications (+ m/z of 305.0682), to cysteines 59 and 377, as well as to the primate sequence-specific cysteine 95 [53] (Fig. 3h, i; data not shown). We concluded from these in vitro and ex vivo experiments that *S-*glutathionylation is a reversible, posttranslational modification of human parkin at select cysteines stemming from exposure to rising concentrations of GSSG under oxidative stress conditions.

### *Prkn* gene expression alters glutathione metabolism in mice

**Fig. 4:**
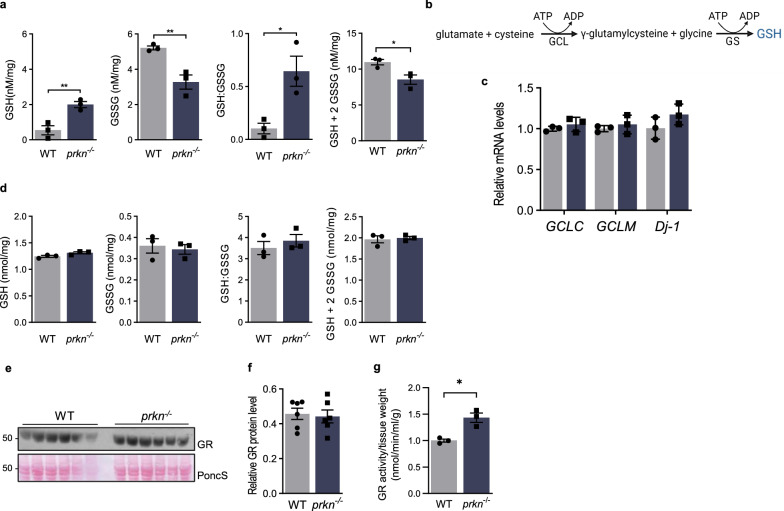
*Prkn* expression alters glutathione metabolism in murine brain, including the activity of glutathione reductase. **a** Tietze method-based quantification of reduced glutathione (GSH), oxidized glutathione (GSSG), the ratio of GSH:GSSG, and total glutathione (GSH + 2GSSG) in mouse brain homogenates of two genotypes, as indicated. **b** Schema of GSH synthesis pathway, where glutamate cysteine ligase (GCL) is the rate limiting enzyme; GS = glutathione synthase. **c** Relative *GCLC, GCLM* and *Dj-1* mRNA levels in the brains of 6 month-old mice (–C and –M denote catalytic and modifying subunits respectively; n = 3 mice/genotype). **d** HPLC-based quantification of GSH, GSSG, the ratio of GSH:GSSG, and the total glutathione pool (GSH + 2GSSG) in brains of 6 month-old mice, as indicated. **e**, **f** Western blot analysis of glutathione reductase (GR) protein levels brains from 6 month-old mice, as separated by SDS/PAGE (reducing conditions) using Ponceau S as the loading control, and its quantification by densitometry (n = 6/genotype) in (f). **g** GR activity in freshly prepared brain homogenates of 6-month-old mice (n = 3/genotype), as indicated. Significance was determined using unpaired Student T-test (a, c, d, f, g) where * represents *p* ≤ 0.05 and **p ≤ 0.01

The impact of *prkn* gene expression on glutathione metabolism and the wider thiol network was also investigated in murine brain. To this end, we quantified GSH and GSSG in WT and *prkn*^*−/−*^ brain lysates by the Tietze method. We found a significant increase in GSH concentrations (*p* < 0.01), decrease in GSSG (*p* < 0.01) and increased GSH:GSSG ratio (Fig. 4a), similar to what was observed in CHO cells above (Additional file 1: Fig. S2). The observed rise in GSH was also consistent with two previous reports using *prkn*^*−/−*^ brain and glial cultures [19, 49]. In exploring the underlying mechanism for this change in glutathione metabolism, we first considered de novo synthesis driven by increased glutamate-cysteine-ligase (GCL) mRNA levels (Fig. 4b); however, we did not detect parkin-dependent changes for the two subunits of this enzyme at the transcriptional level (Fig. 4c).

When we measured GSH concentrations in *prkn*^*−/−*^ murine brains using the HPLC method, we recorded a small trend for a rise in relative GSH levels (and lowering of GSSG; Fig. 4d), as seen by the Tietze method above. The difference in results using these two distinct assays (Fig. 4a vs. d) provided insight into a potentially underlying mechanism: whereas the Tietze method utilizes exogenously added glutathione reductase (GR) to indirectly measure GSH and GSSG levels, HPLC directly measures the net concentrations of GSH and GSSG. Hence, we next examined GR-mediated function as a potential mechanism to explain the relation between *prkn* expression and glutathione’s redox state in murine brain.

### Glutathione reductase activity is upregulated in parkin-deficient mouse brain

We first analyzed whole brain homogenates. Whereas detectable GR protein levels were unchanged in the brains of *prkn*^*−*/−^ mice compared to age-matched (6 month-old) WT controls (Fig. 4e, f), we measured a > 40% increase in GR activity in both freshly prepared and in previously frozen homogenates of *prkn*^*−/−*^ brains when compared to WT animals of the same age (*p* < 0.05, Fig. 4g, Additional file 1: Fig. S3e). The parkin-dependent increase in GSH concentrations and in GR activity was also found in the brains of the bi-genic mice, but not in those from *Sod2*^±^ littermates, thereby suggesting a relatively specific effect induced by parkin deficiency (Additional file 1: Fig. S3a, f).

### Parkin deficiency reveals altered glutathione metabolism in human brain

**Fig. 5:**
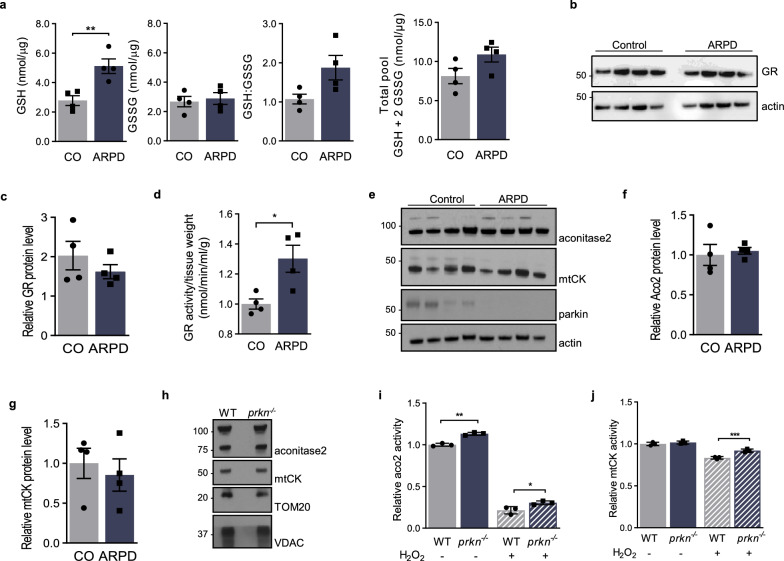
Parkin alters glutathione metabolism in human brain and affects the activity of redox state-dependent enzymes in mice. **a** HPLC-based quantification of reduced glutathione (GSH), oxidized glutathione (GSSG), the ratio of GSH:GSSG, and total glutathione (GSH + 2GSSG) in cortex homogenates from age-matched human control and *PRKN*-deficient autosomal recessive PD (ARPD) cases; **b**, **c** Western blot analysis of glutathione reductase (GR) protein levels, as separated by SDS/PAGE (reducing conditions) **c** its quantification by densitometry (normalized to actin) and **d** GR activity measured in cortex homogenates from the same 8 cases. **e** Western blot results of aconitase-2 (Aco2), mitochondrial creatine kinase (mtCK), and parkin expression in membrane extracts of the four control and four ARPD cortices (as in b–c). **f**, **g** Quantification of relative expression levels of Aco2 and mtCK (shown in **e**). **h** Protein levels of murine Aco2, mtCK, Tom20 and VDAC in mitochondrial extracts from wild-type (WT) and *prkn*^−/−^ brains of 12 month-old mice, as shown by Western blotting. **i** Aco2 and **j** mtCK activities, as measured in freshly isolated mitochondria from WT and *prkn*^−/−^ brains with or without exogenous treatment of 4 μM H_2_O_2_. Data in a-b are plotted as mean values (nmol/µg total protein) ± SEM. Significance was determined using an unpaired Student T-test (a, c, d, f–h) and 2-way ANOVA with Tukey’s post-hoc analysis (i, j), where *p ≤ 0.05, **p ≤ 0.01, and ***p ≤ 0.001

The interplay between parkin and glutathione metabolism was also examined in human brain using frontal cortices from *PRKN*-linked ARPD subjects and specimens from matched controls (Fig. 5a–d) [46, 53]. In the absence of detectable parkin, GSH levels and the ratio of GSH:GSSG were significantly increased, as measured by HPLC (*p* < 0.01 and *p* < 0.05, respectively; Fig. 5a). No differences were seen for GSSG concentrations, nor in the total levels of detectable glutathione in these brains (Fig. 5a). The redox changes recorded in human cortex of ARPD versus control individuals closely mirrored those seen in CHO-control versus CHO-parkin cells under oxidative stress conditions (Additional file 1: Fig. S2) and in adult mouse brain (above). Consistent with the parkin-dependent change observed in murine brain, we saw no difference in GR protein levels in human brain (Fig. 5b, c). However, we measured a significant, ~ 30% elevation in GR activity in lysates from parkin-deficient ARPD cortices when compared to controls (*p* < 0.05; Fig. 5d). We concluded from these complementary results that in mammalian brain a bi-directional crosstalk exists between parkin protein and the metabolism of glutathione.

### Parkin modulates activity of redox state-sensitive, mitochondrial enzymes

To link our redox chemistry findings back to the identified changes from proteomic screens conducted in parkin-deficient mouse brain [36, 38], we explored the activities of select, redox-sensitive enzymes. Although our collective findings pointed at excess oxidative stress in the cytosol of parkin-deficient brain (herein), these unbiased proteomic screens had previously identified dysregulated cytosolic as well as mitochondrial enzymes without apparent difference in mitochondrial structures (or numbers) within neurons of the nigrostriatal pathway [22, 36]. Given that the cellular thiol network, including GSH, involves crosstalk between the cytosol and organelles, we selected two of the dysregulated enzymes, which had been identified by 2D electrophoresis, with important roles in mitochondrial function, i.e., Aco2 and mitochondrial creatine kinase (mtCK) (Fig. 5e–j) [1, 42]. We hypothesized that their redox-sensitive activities could be altered in a *prkn* expression-dependent manner.

Using routine Western blotting, we found no detectable differences in either Aco2 or mtCK protein levels (Fig. 5e–h), nor in several other mitochondrial constituents examined, such as VDAC and MnSOD, in homogenates of cortices from ARPD versus control subjects and in the brains from *prkn*^*−/−*^ mice versus age-matched littermates (Additional file 1: Fig. S4) [54]. In contrast, we recorded a significant increase in the activity of Aco2, even under basal conditions, in mitochondria isolated from *prkn*^*−/−*^ mouse brains when compared to littermate controls (*p* < 0.01; Fig. 5i). Following exposure of freshly prepared brain mitochondria to H_2_O_2_ in vitro, Aco2 and mtCK activities were decreased, as expected; however, their enzymatic functions remained consistently higher in mitochondria from *prkn*^*−/−*^ brains than WT littermates (*p* < 0.05 and *p* < 0.001, respectively; Fig. 5i, j). We concluded from these collective results that the redox state of *prkn*^*−/−*^ mice that includes changes in the thiol network of the adult brain is correlated with differences in the in vitro activities of these two mitochondrial enzymes.

## Discussion

This study builds on our recent discovery that parkin functions as an antioxidant molecule that can neutralize reactive oxygen- and electrophilic species (ROS; RES) in mammalian brain and in vitro [53]. Our goal was to further study redox-linked mechanisms by which the brain responds to parkin deficiency. To this end, we created a mouse model, which combined genetically encoded chronic mitochondrial oxidative stress due to *Sod2* haploinsufficiency with genomic *prkn* deficiency. Age-dependent analysis of these mice revealed that the absence of parkin augmented the degree of redox stress in *Sod2*^±^ mice in the nervous system, including in the midbrain (Figs. 1, 2). This was not sufficient, however, to lead to the degeneration of dopamine neurons in bi-genic animals by 12 months of age [11], which was consistent with a similar approach pursued by others [17]. In exploring possible compensatory mechanisms, we found a heretofore unknown feedback loop between *prkn* gene expression and the regulation of cellular thiols. An upregulation of GSH levels was observed in cells, in human cortex and in mouse brains lacking parkin (Additional file 1: Fig. S3; Figs. 4, 5); as well, we uncovered the direct *S*-glutathionylation of parkin at select cysteines under rising GSSG conditions (Fig. 3). We also provide evidence that parkin’s modulation of the cytosolic redox state impacts mitochondrial function, demonstrated here as an endogenous parkin-dependent change in the activities of two redox-sensitive, mitochondrial enzymes, Aco2 and mtCK. This occurred in the absence of detectable changes in their total protein levels (Fig. 5), or any structural mitochondrial impairment, as studied by others [19, 23, 36, 38].

**Fig. 6:**
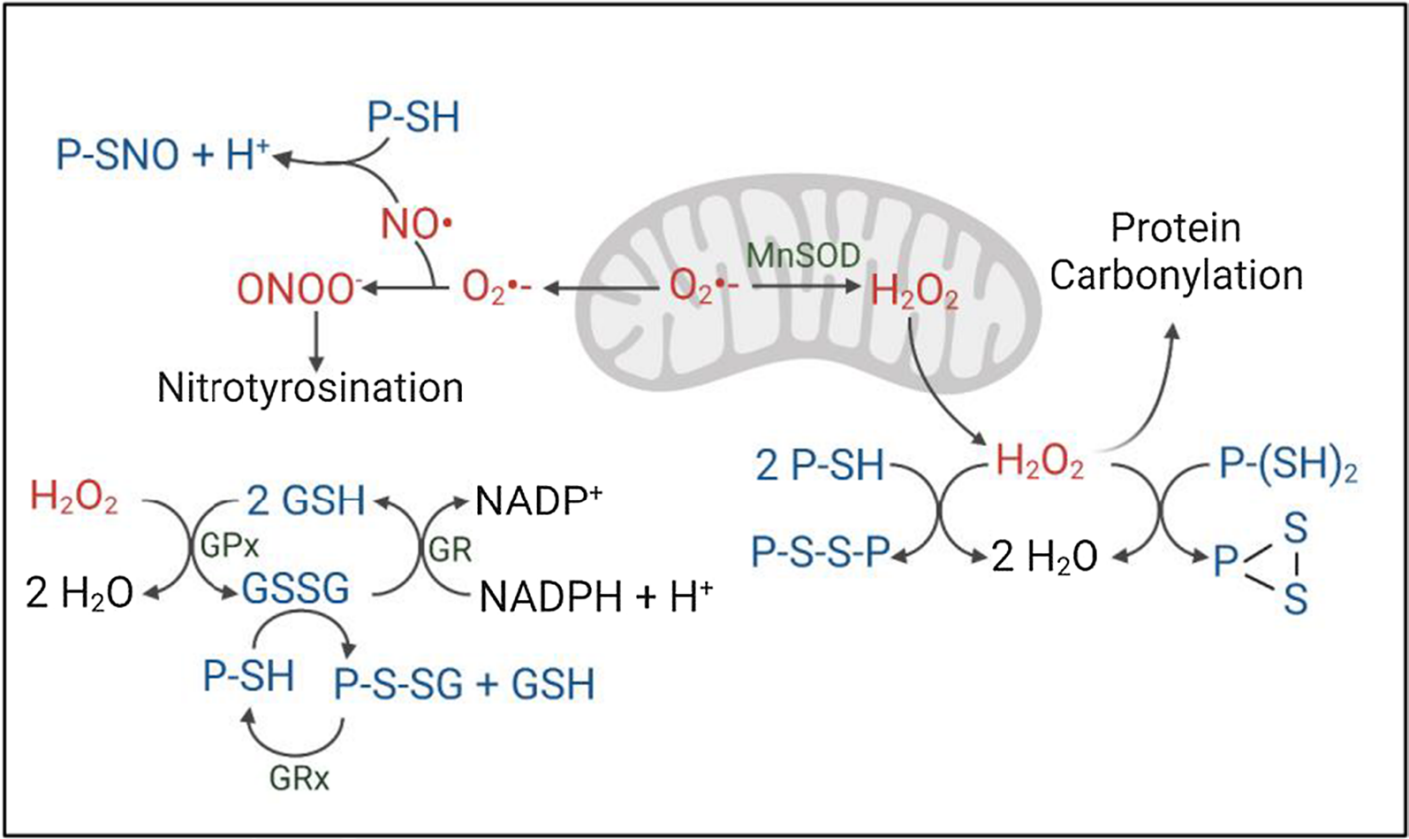
Working model for parkin-dependent effects on the cytosolic redox state in mammalian brain. Graphical depiction of redox changes identified herein, and as published. Highlighted are: GSH recycling; generation of ROS levels (i.e., H_2_O_2_; superoxide) leading to parkin’s variable states of oxidation [53]; protein carbonylation [36]; metabolism of nitric oxide (NO) leading to nitrosylation of parkin and nitrotyrosination of proteins [8, 61]; and function of glutathione reductase (GR), as monitored in normal, mammalian brain. P, parkin; -SH, reduced thiol group; P-*S*-SG, *S*-glutathionylation of parkin; MnSOD, Mn^2+^-dependent superoxide dismutase (SOD2); GPx, glutathione peroxidase; Grx, glutaredoxin

Reduced glutathione is the most abundant cytosolic, low molecular weight thiol involved in antioxidant defenses and in the regulation of cellular metabolism [60]. GSH and the greater thiol network therefore play a role in ageing and the pathogenesis of many diseases, including PD [20, 54]. In the absence of parkin and its many cysteine-based thiols, murine and human brains upregulate GSH recycling, presumably as a compensatory mechanism to keep ROS (and RNS) levels from rising. If done efficiently, it could explain the lack of a significant elevation in endogenous H_2_O_2_ concentrations in parkin-deficient brain under steady-state conditions. Of note, such effective compensation in rodent brain includes the apparent ability to avoid a rise in mitochondrial ROS in *Sod2-*haploinsufficient and bi-genic mice, possibly due to rapid shuttling of O_2_^−^ into the cytosol for processing by alternate SOD activities (Fig. 6). Nevertheless, parkin’s contribution to H_2_O_2_ reduction in the cytosol is unmasked under the following conditions: (i) in the presence of redox stressors, either due to a second genetic hit in mice (Figs. 1, 2); (ii) or caused by a pharmacological agent, as shown in cells (Additional file 1: Fig. S2); or (iii) following a neurotoxicant (e.g., MPTP) administered in vivo in mice; or (iv) as a result of ageing, as shown in human cortices [53]. We found that in mammalian brain, the absence of endogenous parkin is compensated by the following redox indices: an elevation in GSH concentrations; the lowering of GSSG; and an increase in the GSH:GSSG ratio. Of note, the total glutathione concentration (GSH + 2GSSG) remained unchanged, except in the bi-genic mice, where the level was slightly increased (Additional file 1: Fig. S3d). Other teams have previously pointed at altered glutathione metabolism in the context of parkin deficiency, but the underlying mechanisms had remained unknown [19, 49]. We believe that our findings begin to fill this void.

We discovered at least two mechanisms by which parkin modulates glutathione metabolism. First, parkin can directly recycle oxidized glutathione (GSSG) under rising ROS conditions, generating one reduced GSH molecule, which in turn results in the *S-*glutathionylation of parkin itself (P-SH + GSSG $\to$ P-*S-*SG + GSH) (Figs. 3, 6). The latter modification is consistent with our recent report that parkin thiols participate in redox chemistry to neutralize highly reactive molecules*, *e.g., H_2_O_2_ and dopamine metabolites [53], as well as with the literature on transient *S-*glutathionylation of redox-sensitive proteins as a cytoprotective mechanism against rising concentrations of ROS [27]. Our findings are also consistent with parkin’s thiol-based interaction with nitrogen species, as has been published by others [7, 8, 33, 61] (reviewed in: [48]) (Fig. 6).

In the present study, we mapped *S-*glutathionylation of recombinant, human parkin proteins to cysteines 59, 95 and 377. We further demonstrated the redox reversibility of these *S*-glutathionylated residues in parkin by glutaredoxin-1 and -2, but not by thioredoxin-mediated activity (Fig. 3), thereby providing specificity for this oxidative modification [48]. Due to the transient nature of *S-*glutathionylation and the LC–MS/MS protocol employed in our previous work on mammalian brain*,* which had been performed under thiol-reducing conditions [53], we have not yet confirmed the *S*-glutathionylation of endogenous parkin in vivo. However, we noted with interest that the primate sequence-specific cysteine 95 of parkin is targeted for several oxidative events, as shown in our work by *S*-glutathionylation (herein), by H_2_O_2_ and by dopamine radical adduct formation [53]. We speculate that this specific residue, which is located in the linker region of human parkin’s sequence, could play a key role in its folding, in interactions with other molecules, and thus in its functions, akin to the phosphorylation at serine 65 during toxin-induced mitophagy in mammalian cells [3, 30, 34]. We anticipate that oxidative, posttranslational modifications of WT parkin [53], including its *S-*glutathionylation at cysteine 95 (Fig. 3), will inform future structural studies and neuronal culture-based research.

In addition to the possible direct contribution of parkin to GSSG recycling, as shown ex vivo, we identified that parkin also modulates GSSG recycling through the regulation of GR in vivo. We found an increase in GR activity, but not of its protein level, in both parkin-deficient murine and human brain. GR is the cognate enzyme for recycling GSSG back to GSH (Figs. 4, 5, 6); it is activated by rising levels of ROS and/or its substrate, i.e., GSSG. Further, GR is inhibited by an excess concentration of GSH, and by a rise in the NADP^+^:NADPH/H^+^ ratio [12, 13, 52] (Fig. 6). Whether parkin has a direct, negative effect on GR activity, such as through physical binding or, possibly through a form of ubiquitylation not yet detected by us [45], or an indirect effect via the redox state of cellular metabolites (e.g., the ratio of NADP^+^:NADPH/H^+^), remains to be elucidated. Of note, while we cannot completely exclude effects by parkin deficiency on de novo glutathione synthesis, we found no significant rise in mRNA levels of subunits for the rate-limiting enzyme, i.e., glutamate-cysteine ligase (*Gclm* and *Gclc*), nor did we see a consistent rise in total glutathione (GSH + 2GSSG) concentrations across the ‘parkin-deficient-only’ model systems. Our GR activity-related findings may provide a first clue for the mechanism that underlies the heretofore unresolved nature of the ‘intrinsic, pro-mitochondrial effect conferred by parkin’ [4], namely in the thiol network-related regulation of the cell’s redox state, which include important redox-sensitive co-factors, such as the ratio of NADP^+^/NADPH^+^ (Fig. 6) and NAD^+^/NADH^+^.

We posit that the cellular response of increasing glutathione recycling in the absence of parkin’s own thiols is protective [27] and that it likely contributes to the lack of cell loss in the *S. nigra* of genomically *prkn*-deficient mice [15, 19, 37, 38, 47, 57]. The compensation in GSH metabolism was not sufficient, however, to completely protect against a parkin deficiency-linked rise in radicals, as seen by the trend for more nitrotyrosinated proteins and the significant rise in total carbonyl content in the brain (Figs. 1, 2, 6). The latter observation reinforces the concept that parkin cysteines convey polyvalent, antioxidant functions, which synergistically lower stress conferred by ROS, RNS and RES [53]. This parallels antioxidant effects observed for Dj-1 [48].

The strengths of our study include: the usage of multiple, complementary model systems based on endogenous and exogenous *PRKN* expression; the validation of biochemical findings in specimens from human brain; the reproducibility of seminal findings (e.g., parkin’s GSSG-reducing activity) in more than one laboratory by more than one operator and by several different techniques (including those pertaining to changes in glutathione metabolism in vivo); the recent report by Adedara et al., which substantiated many of the biochemical changes that we described here in their *prkn*^−/−^ fly model. Intriguingly, parameters of elevated oxidative stress in parkin-deficient flies and their associated motor deficits were effectively reversed by the antioxidant resveratrol [2]. Last but not least, our findings are indirectly supported in the context of publications on multiple, protective effects by parkin proteins, which the field has produced over the years, that had not yet been mechanistically connected.

Potential weaknesses of our study include: the not yet delineated mechanism by which parkin regulates GR enzyme activity (as discussed above); the omission to characterize the effects of parkin deficiency on glutathione metabolism (and GSSG recycling) in glia versus neurons, as previously initiated by Solano et al. [49]; and the lack of examination of the thiol network in an animal model expressing an E3 ligase-incompetent mutant of human parkin [3, 45, 50, 55]. In future studies, we will also revisit exogenous, dopamine-induced stress to neuronal cell culture models expressing distinct, ARPD-linked *PRKN* genotypes [53] to probe for any differences in their glutathione metabolism.

Further validation of parkin’s multiple effects that contribute to cellular redox homeostasis could create new opportunities in developing urgently needed therapies for patients with young-onset parkinsonism, and possibly, for those with late-onset PD. Finally, redox-based chemical readouts, such as for GR activity in the cerebrospinal fluid, could serve as a possible biomarker for *PRKN* mutation-linked disease, of its progression, and for the response to future interventions [31, 56, 58].

## Supplementary Information

**Additional file 1: Fig. S1.** Wild-type parkin may contribute to lowering of oxidative stress in murine hearts.**a** Relative H_2_O_2_ concentration and **b** total protein nitrotyrosination in heart homogenates of 6 mth-old mice fromthe indicated genotypes. Ponceau S was used as a loading control for relative signal quantification, as shown in **c**(n=3/genotype ± SEM). Significance was tested using 1-way ANOVA with Tukey’s post-hoc analysis (**a**, **c**); nosignificance was found **Fig. S2.**. Parkin over-expression alters the redox state in mammalian cells.** a** Cytotoxicity assayed in CHO cells stably expressing myc-parkin cDNA (denoted as *PRKN+*) or myc-control vectors (denoted as *PRKN-*) under normal conditions with or without the addition of 2 mM H2O2 or 20 mM buthionine sulfoximine (BSO). Data in **a **are plotted as mean normalized to wells of untreated control cells. **b** Relative endogenous H2O2 levels and **c** cellular toxicity in CHO cells stably expressing myc-parkin (*PRKN+*) or myccontrol vector (*PRKN-*) with or without exposure to 2 mM H2O2, 2 mM BSO, or 20 mM N-acetyl cysteine (NAC), as indicated. **d**, **f** HPLC-based quantification of reduced glutathione (GSH), oxidized glutathione (GSSG), the ratio of GSH:GSSG, and total glutathione pool (GSH+2GSSG) in CHO cells under control conditions (**d**), and following H2O2 stress (**f**). **e** Quantification of GSH by monochlorobimane assay (Tietze method) in SH-SY5Y neural cells transiently over-expressing FLAG-parkin (*PRKN+*) or FLAG-control vector (*PRKN-*). Results were obtained using 3 independent experiments ± SEM. Statistical significance was determined using a One-sample T-test with each column compared to 1.0 (**a**; not significant), 2-way ANOVA with Tukey’s post-hoc (**b**, **c**) and unpaired Student T-test (**d**–**f**), where *p ≤ 0.05, **p ≤ 0.01, and ***p ≤ 0.001, as indicated **Fig. S3.**. *Prkn *expression alters glutathione metabolism in murine brain. **a**–**d** Quantification of reduced glutathione (GSH), oxidized glutathione (GSSG), their ratio, as well as the total concentration of glutathione in brain homogenates from 6 mth-old mice, as quantified by HPLC, for the indicated genotypes. **e **Glutathione reductase (GR) activity in homogenates of previously frozen brain from 7 to 8 mth-old WT and *prkn*^−/−^ mice (n = 6, mean normalized to WT ± SEM). Significance was determined using an unpaired Student T-test with *p* = 0.029. **f **GR activity measured in freshly prepared brain homogenates of 6 mth-old mice from 4 different genotypes (as in panels a-d; n = 3/genotype). Significance was determined using a 1-way ANOVA with Tukey’s post-hoc analysis (**a**–**d**, **f**) and unpaired Student T-test (**e**) with *p ≤ 0.05 and **p ≤ 0.01 **Fig. S4.** Western blot results for redox state-related proteins in fractionated human cortices.** a** Specimens from matched control and human ARPD (*PRKN *mutant) frontal cortices, as described above (and in: Shimura H et al. 2001; Tokarew J et al., 2021), were serially fractioned using increasing concentrations of detergent from readily saline-soluble (TS) state, to lipid-bound, TX100-soluble (TX) state and insoluble (SDS) state, as described in Tokarew J et al., 2021. Fractions were analyzed by SDS/PAGE under reducing conditions and immunoblotted with antibodies to parkin, DJ-1, voltage-dependent anion channel (VDAC), manganese superoxide dismutase (MnSOD) and glyoxalase-1, and membranes counterstained with Ponceau S, as indicated.

## Data Availability

Original data associated with this study are available in the main text and supplementary figures and tables; additional data will be made available upon request.
