# Supplementary material for: Parkin coregulates glutathione metabolism in adult mammalian brain
Source: Acta Neuropathol Commun. 2023 Jan 23;11:19. doi: 10.1186/s40478-022-01488-4 (PMC9869535; doi:10.1186/s40478-022-01488-4)
Supplement: Supplementary file 1 — Additional file 1: Fig. S1. Wild-type parkin may contribute to lowering of oxidative stress in murine hearts.a Relative H2O2 concentration and b total protein nitrotyrosination in heart homogenates of 6 mth-old mice fromthe indicated genotypes. Ponceau S was used as a loading control for relative signal quantification, as shown in c(n=3/genotype ± SEM). Significance was tested using 1-way ANOVA with Tukey’s post-hoc analysis (a, c); nosignificance was found Fig. S2.. Parkin over-expression alters the redox state in mammalian cells. a Cytotoxicity assayed in CHO cells stably expressing myc-parkin cDNA (denoted as PRKN+) or myc-control vectors (denoted as PRKN-) under normal conditions with or without the addition of 2 mM H2O2 or 20 mM buthionine sulfoximine (BSO). Data in a are plotted as mean normalized to wells of untreated control cells. b Relative endogenous H2O2 levels and c cellular toxicity in CHO cells stably expressing myc-parkin (PRKN+) or myccontrol vector (PRKN-) with or without exposure to 2 mM H2O2, 2 mM BSO, or 20 mM N-acetyl cysteine (NAC), as indicated. d, f HPLC-based quantification of reduced glutathione (GSH), oxidized glutathione (GSSG), the ratio of GSH:GSSG, and total glutathione pool (GSH+2GSSG) in CHO cells under control conditions (d), and following H2O2 stress (f). e Quantification of GSH by monochlorobimane assay (Tietze method) in SH-SY5Y neural cells transiently over-expressing FLAG-parkin (PRKN+) or FLAG-control vector (PRKN-). Results were obtained using 3 independent experiments ± SEM. Statistical significance was determined using a One-sample T-test with each column compared to 1.0 (a; not significant), 2-way ANOVA with Tukey’s post-hoc (b, c) and unpaired Student T-test (d–f), where *p ≤ 0.05, **p ≤ 0.01, and ***p ≤ 0.001, as indicated Fig. S3.. Prkn expression alters glutathione metabolism in murine brain. a–d Quantification of reduced glutathione (GSH), oxidized glutathione (GSSG), their ratio, as well as the total concentration o [file 40478_2022_1488_MOESM1_ESM.pdf]

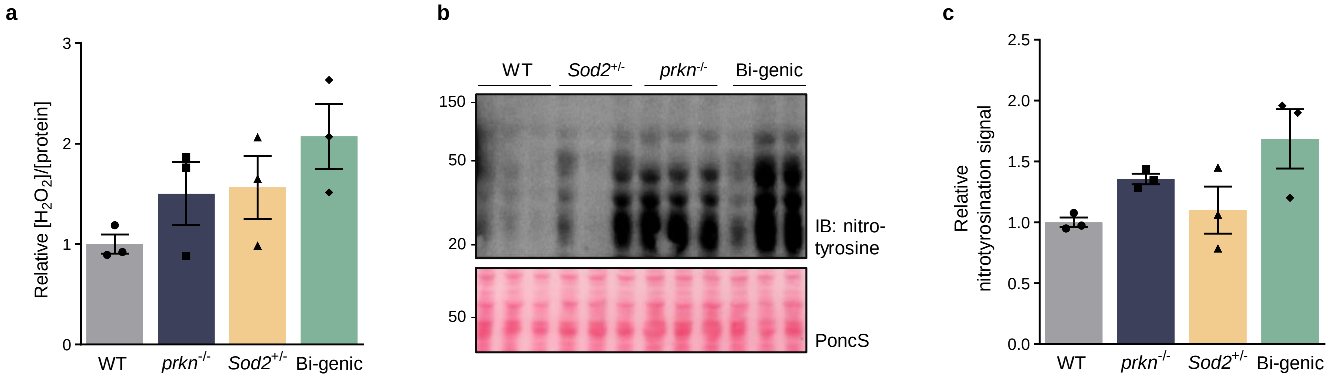

Supplemental Figure 1: Wild-type parkin may contribute to lowering of oxidative stress in murine hearts. **a,** Relative H_2O_2 concentration and **b** total protein nitrotyrosination in heart homogenates of 6 mth-old mice from the indicated genotypes. Ponceau S was used as a loading control for relative signal quantification, as shown in **c** **(**n=3/genotype ± SEM). Significance was tested using 1-way ANOVA with Tukey's post-hoc analysis (a,c); no significance was found.

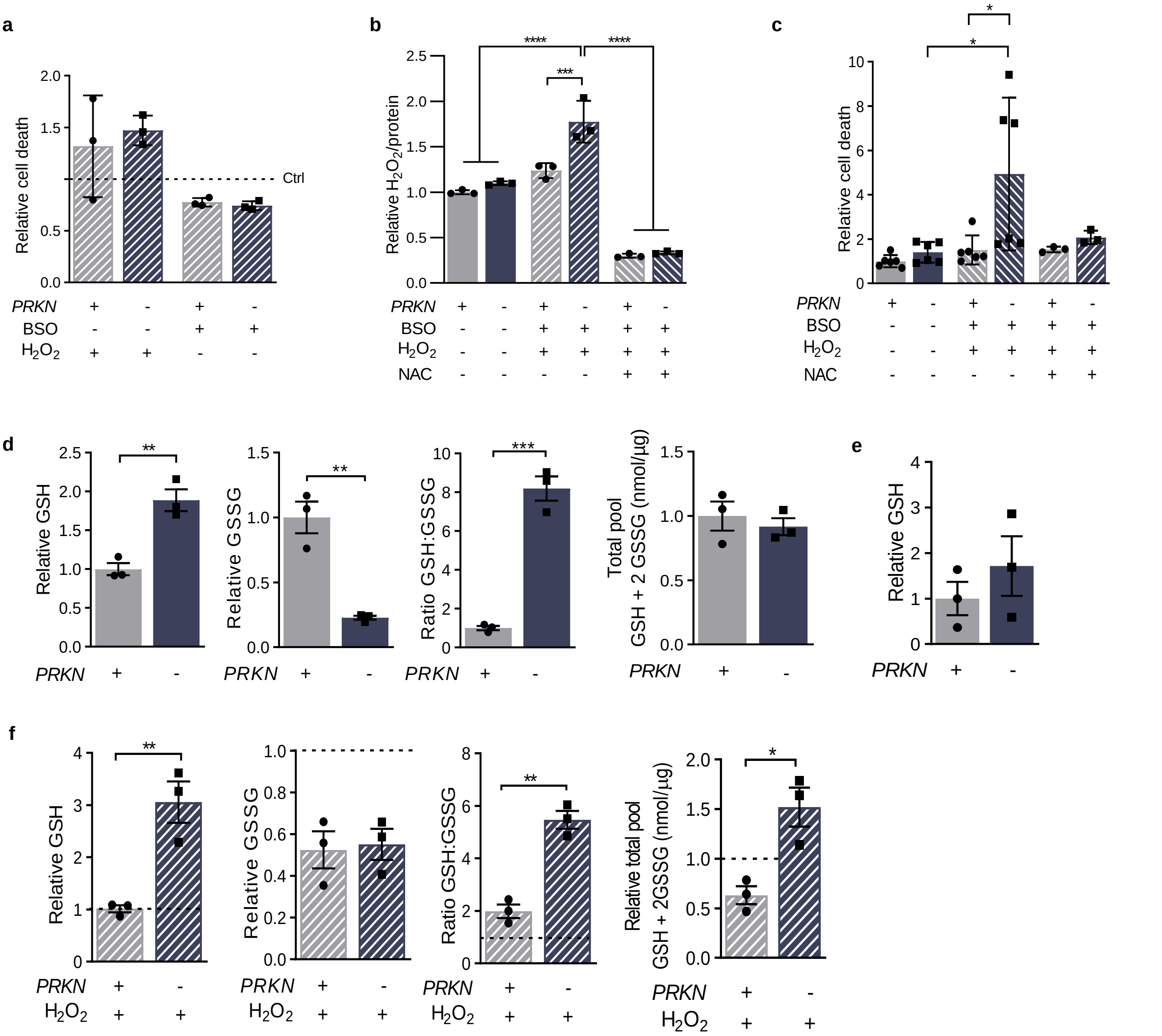

Supplemental Figure 2: Parkin over-expression alters the redox state in mammalian cells. a, Cytotoxicity assayed in CHO cells stably expressing myc-parkin cDNA (denoted as *PRKN*+) or myc-control vectors (denoted as *PRKN*-) under normal conditions with or without the addition of 2 mM H_2O_2 or 20 mM buthionine sulfoximine (BSO). Data in **a** are plotted as mean normalized to wells of untreated control cells. **b,** Relative endogenous H_2O_2 levels and **c,** cellular toxicity in CHO cells stably expressing myc-parkin (*PRKN*+) or myc-control vector (*PRKN*-) with or without exposure to 2 mM H_2O_2, 2 mM BSO, or 20 mM N-acetyl cysteine (NAC), as indicated. **d,f,** HPLC-based quantification of reduced glutathione (GSH), oxidized glutathione (GSSG), the ratio of GSH:GSSG, and total glutathione pool (GSH+2GSSG) in CHO cells under control conditions (**d**), and following H_2O_2 stress (**f**). **e**, Quantification of GSH by monochlorobimane assay (Tietze method) in SH-SY5Y neural cells transiently over-expressing FLAG-parkin (*PRKN*+) or FLAG-control vector (*PRKN*-). Results were obtained using 3 independent experiments ± SEM. Statistical significance was determined using a One-sample T-test with each column compared to 1.0 (**a**; not significant), 2-way ANOVA with Tukey's post-hoc (**b,c**) and unpaired Student T-test (**d-f**), where * $p \leq 0.05$, ** $p \leq 0.01$, and *** $p \leq 0.001$, as indicated.

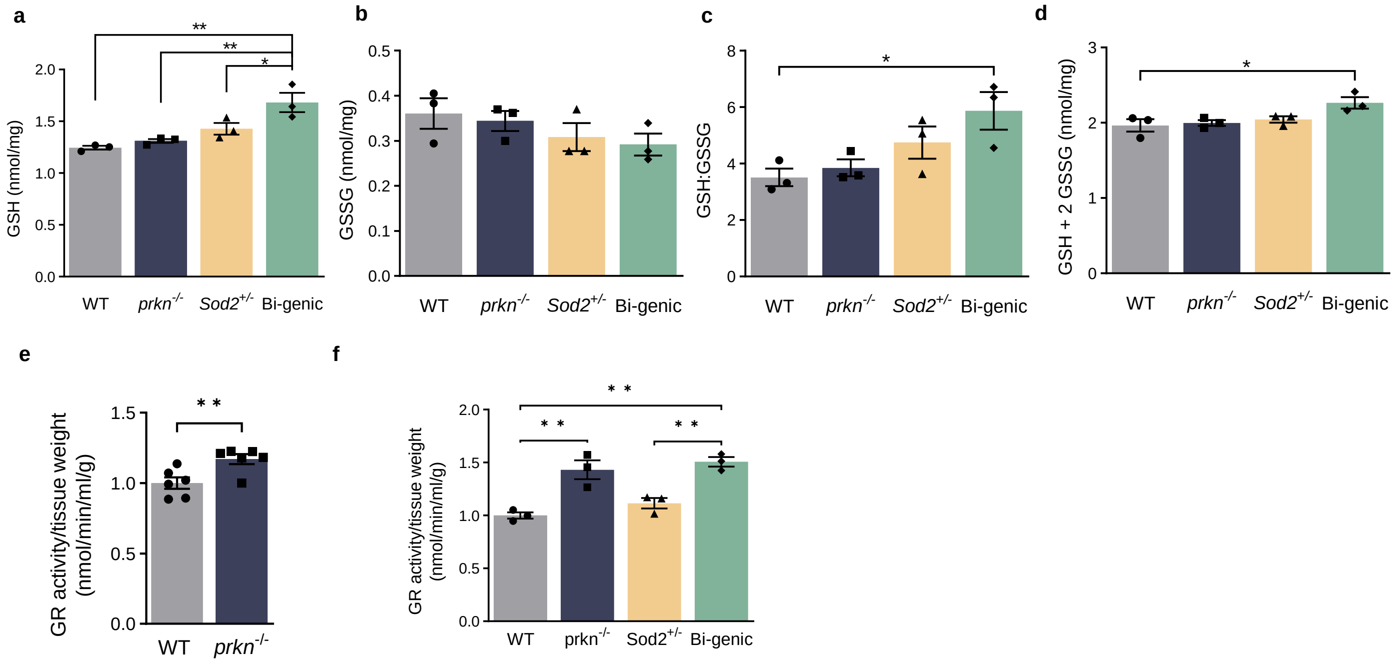

Supplemental Figure 3: *Prkn* expression alters glutathione metabolism in murine brain. a-d, Quantification of reduced glutathione (GSH), oxidized glutathione (GSSG), their ratio, as well as the total concentration of glutathione in brain homogenates from 6 mth-old mice, as quantified by HPLC, for the indicated genotypes. **e,** Glutathione reductase (GR) activity in homogenates of previously frozen brain from 7-8 mth-old WT and *prkn*-/- mice (n=6, mean normalized to WT ± SEM). Significance was determined using an unpaired Student T-test with p=0.029. **f,** GR activity measured in freshly prepared brain homogenates of 6 mth-old mice from 4 different genotypes (as in panels a-d; n=3/genotype). Significance was determined using a 1-way ANOVA with Tukey's post-hoc analysis (**a-d, f**) and unpaired Student T-test (**e**) with * p≤0.05 and ** p≤0.01.

a

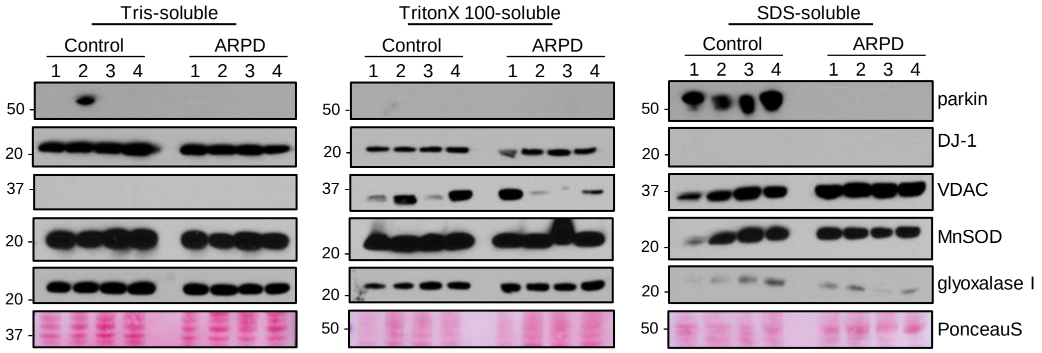

Supplemental Figure 4: Western blot results for redox state-related proteins in fractionated human cortices. a, Specimens from matched control and human ARPD (*PRKN* mutant) frontal cortices, as described above (and in: Shimura H et al. 2001; Tokarew J et al., 2021), were serially fractioned using increasing concentrations of detergent from readily saline-soluble (TS) state, to lipid-bound, TX100-soluble (TX) state and insoluble (SDS) state, as described in Tokarew J et al., 2021. Fractions were analyzed by SDS/PAGE under reducing conditions and immunoblotted with antibodies to parkin, DJ-1, voltage-dependent anion channel (VDAC), manganese superoxide dismutase (MnSOD) and glyoxalase-1, and membranes counterstained with Ponceau S, as indicated.
